# Molecular evolution in light of regulatory-coding epistasis

**DOI:** 10.1038/s44319-026-00821-5

**Published:** 2026-06-10

**Authors:** Pavithra Venkataraman, Christian R Landry

**Affiliations:** 1 Département de biochimie, de microbiologie et de bio-informatique, Faculté des sciences et de génie, Université Laval, Québec, G1V 0A6 QC Canada; 2 Institut de biologie intégrative et des systèmes, Université Laval, Québec, G1V 0A6 QC Canada; 3 PROTEO, Le regroupement québécois de recherche sur la fonction, l’ingénierie et les applications des protéines, Université Laval, Québec, G1V 0A6 QC Canada; 4 Centre de recherche sur les données massives, Université Laval, Québec, G1V 0A6 QC Canada; 5 Centre de recherche en infectiologie, Université Laval, Québec, G1V 4G2 QC Canada

**Keywords:** Evolution & Ecology

## Abstract

Predicting the fitness effects of mutations is central to understanding molecular evolution and interpreting genome sequence data. Such predictions remain challenging due to the inter-dependent roles of coding and non-coding genetic variation. While coding mutations alter protein structure, stability, activity, and sometimes abundance, regulatory mutations modulate gene expression timing and levels. Because coding and regulatory variation are thought to independently impact features of protein function, their combined effects or complex phenotypes are often unexpected. In particular, regulatory-coding epistasis, whereby the fitness effect of a coding mutation depends on the regulatory background, can reshape fitness landscapes and influence adaptive trajectories. In this review, we explore how variation in protein abundance and activity jointly shape fitness, constrain adaptation, and impact molecular evolution. Drawing on examples from systematic studies carried out in unicellular organisms, we speculate on a fitness function integrating abundance and activity and discuss the broader implications of these interactions for evolutionary dynamics, genetic disease, and phenotypic diversity.

## Abstract (web summary)

Coding and regulatory mutations do not act in isolation. This review proposes a conceptual framework for the joint influence of coding and regulatory mutations in shaping fitness, constraining adaptation, and impacting molecular evolution.

## Graphical abstract

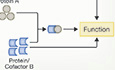

## Introduction

Predicting the fitness effects of mutations helps us anticipate how natural selection may drive adaptation (Gregory, 2009). Mutations occurring in coding regions of the genome can cause changes to the structure, stability, abundance, and molecular interactions of the protein; and their effects can be studied by integrating genomics and synthetic biology with biophysical models (Gelman et al, 2025; McGaugh et al, 2021). These approaches have provided a framework for understanding how amino acid substitutions impact protein function and, consequently, fitness. However, mutations are not confined to coding sequences. Non-coding mutations, including those in regulatory regions, can also have important phenotypic consequences by modulating processes like regulation of transcription and, consequently, protein abundance and function (Buchberger et al, 2019; Katsonis et al, 2014). Tracking the adaptive evolutionary impact of mutations in non-coding regions is challenging, in part because they are highly variable across taxa—in eukaryotes, non-coding regions are vast, but much smaller in bacteria and archaea (Haberle and Stark, 2018; Kuo et al, 2025; Peeters et al, 2013). In most cases, they lack well-defined functional genomic annotations (Guigó, 2023). The effects of non-coding changes are often genetic context-dependent (Brown et al, 2009; McQueen et al, 2025), environment-dependent (Featherstone and Broadie, 2002; Vande Zande and Wittkopp, 2022; Wittkopp et al, 2008; McManus et al, 2010), and in some cases in multicellular organisms, cell-dependent (Scacheri and Scacheri, 2015). Therefore, their effects are difficult to generalize, raising the key question: how do regulatory mutation-induced changes in protein abundance translate into fitness changes? It is well established that insufficient and excessive protein levels can be detrimental (Bolognesi and Lehner, 2018). However, we currently lack a quantitative understanding of the fitness effects and evolutionary contingencies associated with under- or overexpression, as the underlying causes are poorly characterized at a general level across genes and conditions (Moriya, 2015).

**Figure 1:**
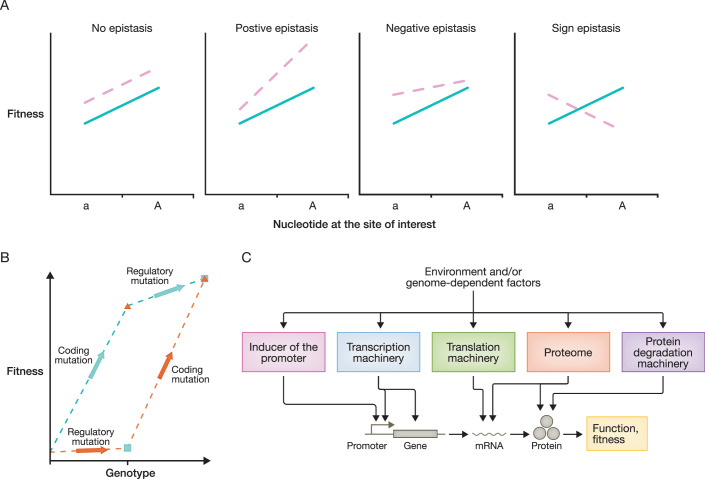
The fitness effects of non-coding mutations depend on the genetic background and the environment. (**A**) This cartoon illustrates how the fitness effect of a mutation (a → A in a specific site) changes across two genetic backgrounds: *b* (solid green line) and *B* (dashed pink line). With no epistasis, the mutation has the same effect in both backgrounds. In positive epistasis, the effect is larger in B than in b; in negative epistasis, it remains beneficial but smaller in B. Sign epistasis represents an extreme case where the mutation is beneficial in b but not in B. (**B**) Example of epistasis between regulatory and coding mutations (inspired by Brown et al, 2009 (Brown et al, 2009)). The regulatory mutation alone provides a small benefit, while the coding mutation gives a larger benefit. However, the regulatory mutation becomes much more beneficial when combined with the coding mutation, illustrating positive epistasis and suggesting sequential fixation of coding followed by regulatory changes. (**C**) While the fitness effects of mutations depend on both the genetic background and the environment, the underlying causes often involve one or more molecular changes. Genomic and environmental alterations can affect the abundance and composition of RNA polymerases, ribosomes, amino acids, and the proteome, including post-transcriptional and post-translational regulators, depending on the organism’s complexity, protein degradation machinery, and other interacting proteins, as well as how the cell imports chemicals from its environment. Together, these changes influence promoter activity, transcription, translation, protein abundance, protein–protein interactions, and ultimately, cellular function and fitness.

Understanding evolutionary changes is also complex due to the fact that the fitness effects of a mutation depend on the broader genetic and environmental context in which they arise. Epistasis, or the interaction between genetic loci, is pervasive (Bateson and Mendel, 1909). Epistasis can occur within a single coding region (Starr and Thornton, 2016), between distinct coding loci (Johnson et al, 2023), within non-coding regions (Kuo et al, 2020; Ang et al, 2023; Bernet and Elena, 2015), between non-coding regions (McQueen et al, 2025), or across regulatory and coding elements (Lagator et al, 2017). Any of these forms of epistasis alters mutational effects in non-additive ways (Fig. 1A), making the fitness effects of combinations of mutations often unpredictable from the effect of single ones (Sandhu et al, 2025). Therefore, in the absence of a mechanistic understanding of how epistasis operates at the cellular level, fully describing evolution requires that the fitness effect of every mutation in every possible genotype is measured in as many environments as possible. However, as the genome length increases, the number of possible mutations increases, and the number of possible genotypic combinations grows exponentially, far exceeding what can be systematically studied in the laboratory or even simulated computationally.

Predictions of fitness changes in microbial evolution experiments have been made through seemingly simple principles. Over the past decade, evolution experiments have shown that the average fitness effect of an adaptive mutation is typically smaller in high-fitness backgrounds than in low-fitness backgrounds, indicating negative epistasis between beneficial mutations in an evolving population, resulting in temporally diminishing returns at the level of fitness (Khan et al, 2011; Chou et al, 2011; Wünsche et al, 2017). This means that, given the fitness effect of a mutation in one background, its effect in other backgrounds can often be predicted without needing to know its precise impact on underlying molecular functions and processes (Kryazhimskiy et al, 2014; Johnson et al, 2019; Ardell et al, 2024; Bakerlee et al, 2022; Diaz-Colunga et al, 2023). Background fitness thus acts as a macroscopic predictor of evolutionary outcomes.

A natural question is whether an analogous, coarse-grained predictor exists at the level of individual proteins. One prominent candidate is protein stability. A growing body of work suggests that a protein’s thermodynamic stability may constrain or bias the trajectory of evolution: destabilized proteins tend to preferentially accumulate stabilizing mutations in selective conditions, and conversely, proteins with excess stability may have access only to destabilizing mutations (Tokuriki and Tawfik, 2009). In this way, protein stability can act as a global constraint on sequence evolution, influencing both the direction and the accessibility of adaptive trajectories.

However, stability, or such a protein property, alone cannot fully explain evolutionary outcomes. For example, a protein-destabilizing mutation does not always lead to disease. This is partly because mutations that affect a protein’s structure or function arise in different regulatory contexts, determined by genetic regulatory elements such as promoters, enhancers, and untranslated regions. Regulatory and coding regions can indeed interact to shape fitness effects. This phenomenon, known as regulatory-coding epistasis, occurs when the fitness effect of a coding mutation depends on the regulatory context, and vice versa (Fig. 1B). These interactions can reshape fitness effects and alter the evolutionary accessibility of genotypes, with far-reaching implications, including for human disease (Brown et al, 2009; Cisneros et al, 2023; McQueen et al, 2025).

Epistatic relationships are additionally complicated by genotype-by-environment (GxE) interactions, which modulate the fitness outcomes of mutations, largely based on the need of the protein they affect (Johannsen, 1911). The environment can act at two levels to modify the fitness of genotypes (Aubé and Landry, 2024). It can alter the mapping between activity, abundance and function, or function and fitness. Generally, changing environments could alter regulation dynamics (Penner-Goeke and Binder, 2024) and hence protein abundance (Munro et al, 2024), metabolic state of the cell (Mallard et al, 2018) and through that, modify the fitness-optimal protein abundance and activity (Dekel and Alon, 2005). Understanding protein evolution, therefore, requires an integrated approach accounting for regulatory architecture governing gene expression, the structural and functional effects of coding mutations, and the genomic and environmental context shaping their interplay (Fig. 1C).

In this review, we examine how variation in protein abundance, together with changes in structure and activity, shapes the evolutionary dynamics of proteins. In this Darwinian context, fitness serves as the most relevant measure of evolutionary success, even though genotype–phenotype relationships are mediated by molecular and physiological intermediates that are often poorly characterized and/or understood. Drawing on evidence from several organisms, we derive insights that inform our understanding of genetic disease, phenotypic diversity, and general principles of molecular evolution.

We begin by reviewing key molecular mechanisms that contribute to how protein abundance is regulated within the cell and how changes in abundance can influence fitness. We then examine the potential costs associated with protein overproduction and the factors that determine optimal protein levels. Next, we explore how coding and regulatory variants interact to shape phenotypes, drawing on examples from various model organisms. We highlight the significance of these interactions in human genetics by exploring some examples of regulatory-coding interactions in humans. We then explain the implications of such interactions for our understanding and predictability of molecular evolution. Finally, we conclude with a perspective that outlines key directions for future research in this area.

## Protein abundance as a fitness determinant

The abundance of individual proteins in a cell can differ by several orders of magnitude (Munro et al, 2024). Yet, even small changes in abundance can significantly impact phenotypes across organisms, from microbes to humans. Such changes, whether environment-induced, caused by spontaneous mutations or synthetically engineered, shape both survival and long-term evolution.

Protein levels can be modified by altering either their production or consumption. Although proteins are not “consumed” in the classical metabolic sense, they transition between conformational states (Bryan and Orban, 2010), and are degraded through pathways that maintain cellular homeostasis. Degradation can be non-specific, as in some lysosomal pathways, or targeted, as in the ubiquitin-proteasome system (Zhao et al, 2022; Cooper, 2000). Mutations in these pathways can therefore cause global, or substrate-specific shifts in protein abundance, depending on which components are perturbed (Chiba and Tanaka, 2005). In yeast, for example, several polymorphisms modulate the activity of the ubiquitin-proteasome protein degradation system, often in substrate-specific ways (Collins et al, 2022). Although we know considerably less about how genetic variation affects degradation rates quantitatively, recent work shows that degradation itself is environment-dependent (Avery et al, 2025). In rapidly growing cells, however, dilution by cell division is often considered to be a major contributor to decreases in abundance of several classes of proteins (Gupta et al, 2024).

In contrast, protein production is governed by transcriptional and translational control, which are directly shaped by both regulatory (non-coding) and coding sequences. Common mechanisms to alter production include changes in gene copy number, transcriptional activity, and translation rates (Kafri et al, 2016). These processes are comparatively well understood, and consequently, most of our knowledge about how genetic variation affects abundance and activity comes from changes in production rather than degradation.

Nevertheless, the same conceptual principles apply to any regulatory step whose rate can be altered by mutations. This framework underlies regulatory-coding epistasis, in which non-coding mutations affect expression and coding mutations alter protein activity, stability, or abundance, and the combined effects interact non-additively to determine phenotypes.

**Figure 2:**
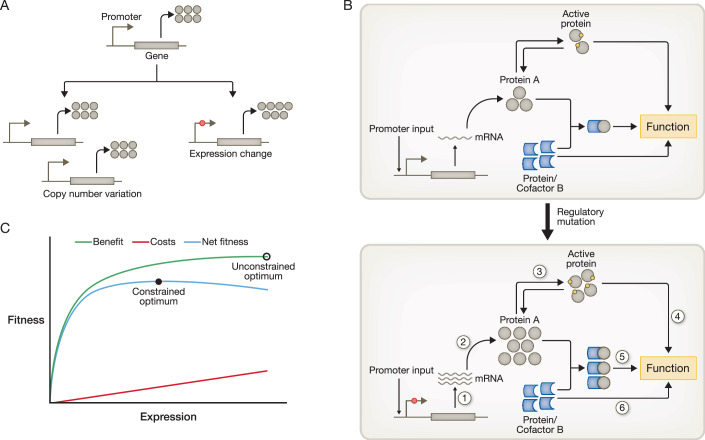
Protein abundance, costs, and evolutionary constraints on expression. (**A**) A promoter–gene pair producing 6 proteins yields 12 upon copy number doubling. In contrast, promoter mutations generally alter expression independently of copy number, enabling finer, non-integral control of protein abundance. (**B**) The top panel illustrates the production of a protein and the downstream processes it is involved in, in a cell. A promoter mutation (denoted by a red circle) that increases expression can impose high costs on the cell. It leads to increased transcription ①, which consumes energy and RNA nucleotides and polymerases, and increased translation ②, which demands additional energy, ribosomes, and amino acids. Once translated, the protein exists in both active and inactive forms, and the transition between these states is energetically demanding ③. These are the direct costs of overexpression (indicated by numbers in continuous red circles). The active form performs its primary cellular function, while the inactive form interacts with another protein to carry out a secondary role ④. Since protein A is overproduced, it could sequester the cofactors that it binds with to perform cellular function ⑤. This sequestration of the cofactor could result in a decrease in the function that the cofactor individually performs ⑥. The excess accumulation of the different forms of the protein and their associated complexes can overwhelm the cell’s protein quality control systems, disrupting protein homeostasis. Additionally, the downstream stoichiometric changes may have fitness costs. These types of non-energetic, indirect costs are indicated in discontinuous circles in the figure. (**C**) The net fitness resulting from overexpression reflects the balance between its benefits and costs. When fitness effects saturate with expression, as shown by the green curve, and the cost function is linear, as shown by the red line, the resulting net fitness follows the blue curve. Without any cost of overexpression, the optimal expression level would be higher than when such costs are present.

In the following section, we examine diverse examples from laboratory and natural systems of variations in protein abundance that impact fitness (Fig. 2A). We also explore the cellular costs associated with elevated protein levels (Fig. 2B) and how these trade-offs constrain the evolution of gene expression (Fig. 2C).

### Fitness consequences of copy number variations

Alterations in protein levels can arise rapidly (single mutational event) through changes in gene copy number, a class of genetic changes known as copy number variations (CNVs) (Fig. 2A). CNVs involving duplications (or deletions) of genomic regions comprising protein-coding genes have been observed across taxa (Pös et al, 2021) and generally increase the mRNA and protein abundance of the corresponding genes. One of the most widely studied adaptive phenotypes associated with CNVs is stress response. For instance, in plants, CNVs have been shown to confer herbicide resistance (Gaines et al, 2010). In microbes, where evolutionary and molecular mechanisms are often studied in detail, CNVs underlie resistance to chemicals (von Rozycki and Nies, 2009; Chow et al, 2012; Adamo et al, 2012; Fijarczyk et al, 2020) and antibiotics (Sandegren and Andersson, 2009; Craven and Neidle, 2007; Price et al, 2004; Segovia, 1994; Mary et al, 2010; Soo et al, 2011; Selmecki et al, 2006; Sionov et al, 2010; Ni et al, 2013; Berman and Krysan, 2020). CNVs also contribute to adaptation in nutrient-limiting (Harari et al, 2018; Gresham et al, 2008, 2010; Brown et al, 1998), osmotic (Dhar et al, 2011), thermal stress conditions (Riehle et al, 2001; Christ and Chin, 2008), and are thought to contribute to variation in ecological strategies (Klappenbach et al, 2000; Raj and Saini, 2024). In most of these cases, fitness benefit is attributed to the increased protein dosage.

In humans, one of the most noted examples of adaptation via CNVs comes from the genes that code for α-amylase enzymes. All three genes, AMY1 (salivary), AMY2A, and AMY2B (pancreatic), show variable copy numbers across individuals that correlate with the amount of starch in the diet of the populations (Groot et al, 1989, 1990). While the reference genome (GRCh38) contains three copies of AMY1, copy numbers can range widely, especially for AMY1. In contrast, other great apes lack such variation, typically carrying a single copy of each gene. These genes arose through ancestral duplications, but recurrent CNVs are unique to modern humans, absent in Neanderthals and Denisovans (Inchley et al, 2016). This makes the amylase locus a curious case of rapid structural evolution, likely shaped by dietary shifts (Pajic et al, 2019).

CNVs have also been identified as causes of genetic diseases. One of the most well-known CNV-related conditions is Down’s syndrome, caused by trisomy of chromosome 21 (Lejeune et al, 1959). CNVs of smaller regions have been associated with traits such as thalassemia (Higgs et al, 1979) and color blindness (Nathans et al, 1986). More recent and exhaustive studies show that the overall burden of CNVs negatively affects approximately 40 human traits, including kidney, liver, and brain function (Auwerx et al, 2022, 2024).

CNVs are generally expected to affect protein abundance in multiples of single-copy gene expression. For instance, gene duplications often lead to a doubling of the protein product (Moriya, 2015). However, exceptions are common, depending on their effects on transcript abundance (Cardoso-Moreira et al, 2016). In *Drosophila melanogaster*, tandem duplicates of the *Adh* gene showed more than double the expected expression due to transcriptional overactivity (Loehlin and Carroll, 2016). Conversely, in budding yeast, posttranslational mechanisms can buffer protein levels despite increased gene dosage across large segments of the genome (Dephoure et al, 2014) or individual genes (Ascencio et al, 2021). Thus, while copy number changes typically scale protein abundance, deviations from proportionality are not uncommon.

Apart from CNVs, protein abundance can also be increased through transcriptional upregulation. These gene expression changes may enable finer tuning of protein abundance compared to copy number variations (Fig. 2A), depending on the effect sizes of individual regulatory mutations, which remain to be examined for most genes. In the next section, we explore examples of phenotypic changes resulting from altered gene expression.

### Fitness consequences of gene expression changes

One powerful way of examining the consequences of increased protein abundance is using overexpression screens, which were first carried out more than 40 years ago. These screens involve the artificial upregulation of gene expression. Such approaches have been used to identify potential drug targets (Rine et al, 1983; Bharucha and Kumar, 2007; Luesch et al, 2005) whereby a more abundant drug target is expected to lead to resistance to the drug. Additionally, overexpression screens have also helped identify exogenous *E. coli* genes, which, when upregulated, rescue cells with metabolic auxotrophies (Patrick et al, 2007). On the other hand, evolution experiments revealed candidate genes that, when upregulated, improve the growth of microbes under nutrient-limiting conditions (Venkataraman et al, 2024; Morin et al, 2020), and in salt (Gaxiola et al, 1992; Gläser et al, 1993; Ferrando et al, 1995; Mulet et al, 1999; Mendizabal et al, 1998; Sasaki et al, 2017) and metal ion stresses (Daran-Lapujade et al, 2009; Conklin et al, 1992; Kamizono et al, 1989; Zeng et al, 2023). Expression changes have also been associated with adaptive cell-cycle changes in yeast, providing a stepping stone towards larger phenotypic and fitness changes. For instance, adaptive evolution experiments using laboratory yeast populations have shown that alterations in the expression of the spindle body genes is more likely to cause diploidization (Jaspersen et al, 2002; Sing et al, 2018), which helps overcome nutrient, osmotic, and temperature stresses (Harari et al, 2018; Dhar et al, 2011; James et al, 2008; Gresham et al, 2008, 2010; Brown et al, 1998). Additionally, the repression of essential cell-cycle genes results in fitness improvement in optimal growth conditions (Conti et al, 2022). Similarly, non-coding variants of the *SFL1* gene showed strong, strain-dependent epistatic effects on fitness by modulating flocculation (Ang et al, 2023). In a similar study, it was shown that natural yeast variants with environment-dependent effects cluster in promoters, frequently overlapping transcription factor binding sites (Chen et al, 2023). In the plant *Arabidopsis thaliana*, overexpression of a GTP-binding protein enhanced resistance to the pathogen *Pseudomonas syringae* DC3000, widely known for causing disease in tomato plants (Xu et al, 2021). In mice, the overexpression of STIL, a gene involved in cell-cycle regulation, reduces the likelihood of tumor formation, but at the cost of reduced lifespan, indicating that overexpression may have constraints that result in fitness “costs” (Moussa et al, 2023).

For essential genes, the relationship between fitness and expression, obtained by measuring fitness at different expression levels, is typically concave, as illustrated by the green line in Fig. 2C (Keren et al, 2016; Moriya, 2015). This implies that fitness does not increase uniformly with expression and that it will eventually decrease because expression often incurs costs that outweigh the benefits. In the next section, we explore examples of such fitness costs resulting from altered protein abundance, examine the underlying cellular mechanisms, and consider how evolution may have shaped genetic changes to mitigate these burdens.

### Costs of protein overabundance

Protein expression costs, in this context, refer to the direct or indirect negative effects associated with increased protein abundance (summarized in Fig. 2B). Direct costs stem from the energetic burden of producing proteins. RNA and protein synthesis consume significant resources, including ATP and amino acids (Kafri et al, 2016; Davison, 1966; Waldron and Lacroute, 1975). When unnecessary proteins are expressed, these resources are diverted from essential cellular functions, potentially leading to reduced growth or competitive fitness.

Indirect costs arise from unintended perturbations to cellular networks. Overabundant proteins can titrate out shared ribosomes, cofactors, or binding partners; interfere with regulatory interactions, or mislocalize and misregulate downstream targets. For example, overexpressed transcription factors bind off-target DNA sites or sequester co-regulators, thereby altering global gene expression (Deniaud et al, 2009). Similarly, excess metabolic enzymes can distort metabolic fluxes, deplete substrates, generate toxic intermediates, or alter the stoichiometric balance of different folding states of the protein assemblies (Moriya, 2015; Bolognesi and Lehner, 2018; Vavouri et al, 2009; Tang and Amon, 2013; Makanae et al, 2013; Birchler and Veitia, 2012; Bolognesi et al, 2016; Adler et al, 2014; Geiler-Samerotte et al, 2011; Pal et al, 2026; Tomala and Korona, 2013). Chronic overexpression may also activate stress responses such as the unfolded protein response, further reducing cellular fitness (Eanes et al, 2006; Wortel et al, 2018). This stress could overwhelm quality control systems, resulting in misfolding or aggregation, which is particularly problematic for proteins that are unstable or prone to form inclusion bodies (Stefani and Dobson, 2003; Vavouri et al, 2009; Moriya, 2015). The phenotypic effects of the costs of protein abundance, often due to a combination of mechanisms listed above, have been reported in mice (Sato et al, 1998), flies (Smith and Weiler, 2010; Navarro et al, 2011), yeast (Sopko et al, 2006), and pathogenic microbes (Rajer and Sandegren, 2022).

A combination of direct and indirect costs may result in a cost-expression relationship as depicted by the red line in Fig. 2C. Quantifying the cost of expression alone is challenging, as fitness measurements typically capture the net effect or the balance between benefit and cost. One way to disentangle these components is to examine fitness across a range of expression levels and environments (Yan and Lin, 2025; Kafri et al, 2016). For example, overexpressing a protein in an environment where it is superfluous can reveal the cost of overexpression (Dekel and Alon, 2005). The shape of the net fitness–expression curve can indicate where costs or benefits dominate: plateaus, declines, or steep increases mark regions where fitness is most sensitive to abundance. This approach can also identify thresholds beyond which abundance becomes harmful, and how such limits shift across environments (Keren et al, 2016). These types of experiments revealed that measuring gene expression and fitness at only a single or a few expression levels provides no insights into how phenotype varies across the full expression range, leaving open key questions about the sensitivity of fitness to expression variability.

To fill this gap, fitness–expression relationships must be systematically tested by measuring the phenotype resulting from discrete changes in expression across a broad range. A well-established method to do this is by replacing the native promoter with an inducible one, allowing expression to be finely tuned using varying concentrations of an inducer (Gossen and Bujard, 1992). Studies on the *lac* operon in *Escherichia coli* and the *LCB2* gene in yeast have revealed diverse expression–phenotype relationships: overexpression of some genes shows linear effects on growth rate, while others display threshold-like responses (Dykhuizen et al, 1987; Rest et al, 2013; Dekel and Alon, 2005; Perfeito et al, 2011; Stoebel et al, 2008). While such expression-tuning studies are highly resourceful, they are laborious and focus on a handful of genes and traits. On the other hand, high-throughput parallel studies to identify the fitness effects of expression have helped capture a range of diverse fitness-protein abundance relationships, depending on the environment in which a gene is expressed (Keren et al, 2016). Although the overexpression costs are environment and strain/organism-specific (Sopko et al, 2006; Robinson et al, 2021), a recent genome-wide study in *E. coli* offered a broad perspective into their predictability based on the classes of proteins by showing that overexpression of membrane proteins imposed a much stronger cost on fitness than proteins of other classes upon overexpression (Müller et al, 2025).

Overall, these experiments help identify the underlying fitness–expression relationship, depending on which changes in expression have small, large, positive, or negative effects on fitness. For essential genes, fitness is zero when they are not expressed and increases with expression, until a point beyond which marginal costs outweigh marginal benefits. Therefore, the optimal expression level (corresponding to maximum fitness) in the absence of overexpression costs is expected to be higher than the expression optimum in the presence of expression costs. This results in a decrease in the maximum achievable fitness. Therefore, decreasing expression costs may result in a better match between achieved expression and the optimum amount of protein needed. This is one way by which expression costs constrain the evolution of gene expression (Bédard et al, 2022; Dekel and Alon, 2005).

Since protein abundance levels are modulated by cellular constraints and demands from the environment, a consolidated fitness–expression relationship for a protein that is essential in an environment is likely to resemble the blue line in Fig. 2C, where the optimum expression level of a protein is typically achieved around the wild-type promoter sequence (Keren et al, 2016; Bauer et al, 2015; Brown et al, 2009; Fujita et al, 2024).

### Expression noise and the evolution of regulatory sequences

Generally, the overexpression studies discussed above focus on the average gene expression of a population of cells. However, gene expression is an inherently stochastic process, due to the underlying thermodynamics and Brownian motion of the few molecules involved (Swain et al, 2002). Because of this molecular noise, even isogenic cells from identical environments can differ substantially in their protein levels (Raser and O’Shea, 2005). In the simplest case, expression noise arises from promoters switching between “ON” and “OFF” states, resulting in bursts of transcription. This causes random fluctuations in gene expression levels. Additionally, molecular processes like binding and unbinding of polymerases also contribute to noise by dictating the promoter’s switching dynamics (Kepler and Elston, 2001; Paulsson, 2005). Therefore, noise levels vary among promoters and have been shown to at least partially depend on the gene sequence, the environmental and genomic contexts (Newman et al, 2006; Hornung et al, 2012; Carey et al, 2013; Silander et al, 2012; Wolf et al, 2015; Dong et al, 2011). One would expect this variability to influence fitness interactively with mean expression levels: when the expression level is optimal, noise is likely detrimental to fitness, whereas when the expression level is suboptimal, noise may enable some individuals in the population to achieve optimal fitness. Similarly, if the expression level is above the optimum, larger noise could be tolerated because it may still be possible for some cells to have optimal expression.

There is strong evidence that natural selection has acted to reduce noise in the expression of essential, dosage-sensitive genes (Newman et al, 2006; Batada and Hurst, 2007; Lehner, 2008; Silander et al, 2012; Metzger et al, 2015). However, more recent studies have shown that the fitness effect of noise is highly context-dependent, and selection may have acted to increase noise in some cases (Viney and Reece, 2013; Richard and Yvert, 2014; Liu et al, 2016; Tănase-Nicola and ten Wolde, 2008; Wolf et al, 2015; Saini et al, 2010; Beaumont et al, 2009) as variability among individuals in a population could sometimes be beneficial, for instance, in fluctuating environments. In contrast, high expression noise may lead to suboptimal protein production in a large fraction of the population, resulting in reduced fitness in static environments. It has been shown by experimentally manipulating promoter sequences that in a given environment, if the mean expression of the promoter is close to the optimum as described in Fig. 2C, high expression noise is deleterious, but becomes beneficial as the mean expression becomes suboptimal (Duveau et al, 2018). Therefore, noise, apart from overexpression costs, imposes an additional constraint on the evolution of promoter sequences (Lehner, 2008; Metzger et al, 2015).

These observations suggest an important pattern: the evolutionary constraints acting on gene expression are not limited to achieving an optimal mean for fitness, but also extend to minimizing or, in some cases, maintaining noise depending on environmental demands closely tied to the proteins they control. Therefore, promoter sequences and their cognate coding regions must coevolve to maintain expression levels and protein function within a fitness-optimal regime that includes noise as a dimension. In the next section, we examine the molecular mechanisms that underlie these interdependencies and how this interaction influences the coevolution of promoter–gene pairs.

## Mechanisms of interactions between coding and non-coding regions and examples of their compensatory coevolution

Fitness depends on both protein activity and abundance, with the latter often being regulated by non-coding sequences. Because mutations can affect either or both of these features, evolution tends to favor combinations of mutations whose joint effects are compatible with the fitness optimum. Several studies have shown that coding and regulatory mutations often interact non-additively to determine fitness.

One of the earliest and most striking examples of such regulatory-coding epistasis comes from the evolution of β-lactam resistance in *E. coli*: a four-mutation allele of the β-lactamase (*bla*) gene, combined with a regulatory mutation that increases its expression, results in a ~100,000-fold increase in resistance to cefotaxime (Weinreich et al, 2006). The effect of the overexpression mutation depended strongly on the protein sequence: catalytically more efficient alleles showed greater increases in resistance upon overexpression. Thus, the fitness impact and the order of the regulatory mutation depended on the coding sequence it upregulated (Brown et al, 2009), suggesting that promoter–gene pairs coevolve and jointly define fitness. The importance of proper promoter–gene matching is further highlighted by cases where mismatched regulatory and coding sequences incur fitness costs. In *Bacillus subtilis*, the two glutamate dehydrogenase genes, *gudB* and *rocG*, encode enzymes with similar activity but different regulation: *gudB* is constitutively expressed, while *rocG* is tightly controlled. Swapping their promoters disrupts fitness (Noda-Garcia et al, 2017). Protein analyses reveal that the *gudB* enzyme is active only at high glutamate concentrations, reflecting tight regulation of enzymatic activity, whereas the *rocG* enzyme remains active even at low glutamate levels, indicating weaker regulation. Thus, promoter–gene swaps cause metabolic imbalance: either excessive glutamate degradation when a constitutive enzyme is driven by a constitutive promoter or insufficient activity when both enzyme and promoter are tightly regulated. This example illustrates the coevolution of coding sequences and their regulatory elements.

**Figure 3:**
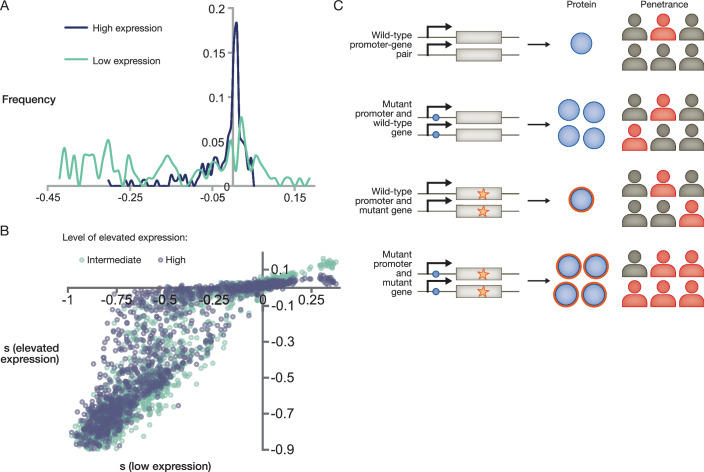
Regulatory-coding epistasis and its effect on disease risk. (**A**) The fitness effects of a library of single amino acid mutations in yeast Hsp90 were measured across different expression levels. The figure (based on the average fitness effect data from Jiang et al (2013) shows the distribution of fitness effects (DFE), which describes the range and likelihood of fitness consequences of new mutations (*s*). The DFE clearly shifts with expression: higher expression levels, by increasing transcription, buffer the deleterious effects of some mutations but also reduce the benefits of others, resulting in a narrower distribution. (**B**) A similar, more comprehensive deep-mutational scanning study of the *dfrB1* gene in *E. coli* further highlights the influence of expression on mutational effects. The figure (based on the average fitness effect data from Cisneros et al, (Cisneros et al, 2023) compares fitness effects at high or intermediate expression levels (promoter activity) with fitness at low expression. Increased expression buffers mildly deleterious mutations but also reveals the costs of overexpression, as beneficial mutations show reduced fitness gains at higher expression levels. (**C**) This example (inspired by Castel et al (2018) illustrates how regulatory and coding mutations interact to produce disease in a heterogeneous population. In the wild type, the gene is minimally expressed, resulting in low disease penetrance. A homozygous regulatory mutation increases transcription, producing more protein and increasing penetrance, similar to a homozygous deleterious coding mutation that is also minimally expressed. However, when both mutations are homozygous, expression of the mutant protein causes disease in most individuals. Thus, regulatory mutations can non-additively modulate the penetrance of disease-causing coding variants.

Although such examples support the existence of regulatory-coding epistasis and the coevolution of promoter–gene pairs, uncovering generalizable principles requires systematic approaches. Deep-mutational scanning (DMS) has emerged as a powerful tool for quantifying the fitness landscape of a locus in a high-throughput and reliable manner (Fowler and Fields, 2014). While it has mainly been used to study coding mutations, some studies have coupled a library of coding mutations with different expression strengths to elucidate the evolutionary constraints on coding and non-coding sequences. In these contexts, modifying expression levels reflects the potential effects of mutations on expression levels. For example, in a mutational scan of the yeast heat shock protein Hsp90, the fitness effects of amino acid changes were shown to exhibit negative epistasis with expression level modulated by different promoters. At high expression, functionally deleterious effects of many mutations were buffered, whereas the same mutations were deleterious at lower expression levels (Jiang et al, 2013) (Fig. 3A). Similarly, a comprehensive study in *E. coli* explored the fitness effects of all single amino acid changes in the *dfrB1* gene across multiple expression levels using an inducible promoter. At suboptimal promoter activity for the WT enzyme, mutations that enhanced protein stability or abundance were generally beneficial. However, at higher expression, these same mutations offered diminished returns with a slight cost at higher expression. Moreover, slightly destabilizing coding mutations could be compensated for by increased expression (Fig. 3B). These findings illustrate compensatory coevolution between promoters and genes, due to regulatory-coding epistasis, where mutations in one locus can buffer, mask, or expose the fitness effects of mutations in the other (Cisneros et al, 2023). At the physiological scale, DHFR function is coupled to thymidylate synthase (TYMS), whose activity must remain below that of DHFR to maintain folate balance (Schober et al, 2019). At low expression of *dfrB1*, TYMS activity may exceed DHFR’s, especially for loss-of-function variants, leading to the depletion of reduced folates. So mutations that increase the abundance of such variants are beneficial. At higher expression, on the other hand, folate imbalance is likely alleviated. Therefore, further increases in protein abundance beyond this point are expected to be costly, and hence the same coding mutations may now have different fitness effects.

Genomic analyses lend further support to the dependence of the fitness effects of coding mutations on expression levels. Evolutionary rate studies have shown that highly expressed genes tend to evolve more slowly than genes expressed at lower levels (Martincorena et al, 2012; Stefani and Dobson, 2003; Jayaraman et al, 2022; Bédard et al, 2022; Drummond et al, 2005). Initially, this was attributed to the essential nature of highly expressed genes and their centrality in protein–protein interaction networks, making mutations in them highly deleterious (Pál et al, 2001). However, subsequent work has proposed that high expression selects for coding sequences that are robust to translational errors, favoring amino acid choices that reduce misfolding risks. Accordingly, the relationship between expression and evolutionary rate reflects selection for protein stability and translational accuracy, rather than functional importance per se (Drummond et al, 2005). Experimental support for the misfolding avoidance hypothesis came from a study in yeast using GFP and URA3 as model proteins. This work showed that the fitness effects of coding mutations depend not only on their biochemical properties but also on the level of gene expression and the environmental context. Mutations that might be tolerated at low expression could become harmful when expression is high, particularly under stress or when misfolded proteins accumulate (Wu et al, 2022). Accordingly, overexpression may aggravate the deleterious effects of some coding variants. In this view, expression level modulates the deleteriousness of amino acid substitutions even over evolutionary timescales. However, it must be noted that the DMS study carried out on *dfrB1* in *E. coli* did not make the same observations (Cisneros et al, 2023), suggesting that these effects could depend on the properties of the proteins studied.

Overall, the coupling between regulatory and coding sequences has led to the evolution of intricate compatibility between promoters and their cognate genes, reflecting a broad evolutionary principle in which both regulatory and coding changes shape phenotypes in a coordinated manner. These relationships are embedded within a cellular context that includes posttranslational surveillance and stress response systems.

### Relevance to human genetics

The consequences of regulatory-coding epistasis are not confined to microbes or model organisms; they are increasingly recognized as central to understanding genetic variation and disease in humans (Milne and Antoniou, 2011; Cooper et al, 2013; Castel et al, 2018; Emison et al, 2005). This interdependence can explain patterns of variable penetrance, context-specific effects, and the buffering or exacerbation of deleterious mutations. Building on the evolutionary logic discussed earlier, we now explore how variation in regulatory regions and their interactions with coding variants manifest in the human genome, and how they contribute to phenotype and disease susceptibility.

In 1975, King and Wilson proposed that most of the morphological differences between humans and our closest relative, the chimpanzee, stem from changes in non-coding regions, rather than coding ones (King and Wilson, 1975). Their work using blood proteins was one of the earliest to demonstrate the importance of studying the evolution of regulatory mechanisms in humans, as protein sequences appear to evolve too slowly to explain human specificity. Non-coding mutations in the human genome, like in other organisms, contribute to phenotypes by altering gene expression through the modulation of transcription factor binding, alterations to gene expression patterns, and post-transcriptional modifications (Peña-Martínez and Rodríguez-Martínez, 2024; Stranger and Dermitzakis, 2005; Cheng et al, 2024; van der Lee et al, 2020). An early example of the role of regulatory mutations in disease phenotypes comes from a 1982 study, which reported that single-nucleotide polymorphisms (SNPs) in the promoter of the β-globin gene were linked to β-thalassemia (Orkin et al, 1982). Since then, and in the post-genomics era, it has been challenging to determine which of the millions of mutations in the enormous non-coding regions of the human genome are responsible for phenotypes. The Encyclopedia of DNA Elements (ENCODE) consortium was established in 2004 to address this challenge. The initial findings of this project suggested that about 80% of the genome has some biochemical activity, and previously understudied regions such as enhancers, silencers, and untranslated regions play regulatory roles (ENCODE Project Consortium, 2004). While subsequent analyses have revised the estimated functional fraction of the genome downward (Graur, 2017; Christmas et al, 2023), genome-wide association studies have revealed that over 90% of disease- and phenotype-associated variation originates from non-coding regions (Peña-Martínez and Rodríguez-Martínez, 2024; Lee et al, 2018; Maurano et al, 2012; Buniello et al, 2019; Deplancke et al, 2016; Zhang and Lupski, 2015). Recent estimates indicate that 90% of mutations in enhancers are neutral, and approximately 30% of mutations in core promoter regions are deleterious (Di et al, 2025).

The phenotypic consequences of regulatory changes among these non-coding variants likely depend on the properties of the coding sequences affected because of the reasons mentioned above. As demonstrated in other organisms, such regulatory-coding epistasis is likely to play a significant role in shaping phenotypic variation and disease risk in humans.

A genome-wide association study reported in 2004 was one of the first to study the combined effects of regulatory and coding variation in humans. Using the genotype and gene expression data obtained from about 200 individuals, the findings of this study showed that about 18% of the coding mutations exhibited phenotypes that were significantly dependent on the *cis-*regulatory variant (Dimas et al, 2008). Functionally, the findings of this study highlight that *cis-*regulatory variation can mask or amplify the phenotypic effects of protein-coding variants. Another study carried out using population-scale human genome resequencing and RNA sequencing data reported that highly expressed coding regions have fewer variants, indicating that highly expressed genes may be under purifying selection that prevents the accumulation of deleterious mutations. Additionally, this study hypothesized that disease states may be altered due to interactions between regulatory and coding variants in the genome that alter the penetrance of coding mutations (Lappalainen et al, 2011). Empirical evidence of altered penetrance due to changes in expression has been reported from clinical studies. For instance, mutations in the low-density lipoprotein receptor (LDLR) cause hypercholesterolemia. In a patient with myocardial infarction who was resistant to conventional lipid-lowering treatments, a mutation in the LDLR promoter that increased the expression of the mutant protein resulted in a severe disease phenotype (Snozek et al, 2009). Similarly, mutations in the gene *KCNQ1* cause type I long QT syndrome, often leading to life-threatening arrhythmias. Allele-specific effects on disease severity exhibited by identical coding mutations were explained by the SNPs in the 3’ UTR region (Amin et al, 2012). Mutations in the thyroid receptor β gene (*THRB*) lead to resistance to thyroid hormone (RTH), and one of the most frequent ones among them is R338W. Patients with this mutation present a wide range of symptoms, suggesting that various factors, in combination with the mutation, contribute to the disease presentation in the clinic. In vitro characterization of the non-coding region of the gene revealed that a SNP in the intron enhancer region of the *THRB* gene leads to the overexpression of the mutant allele, tilting the expression balance between the WT and the mutant allele in a tissue-specific fashion, and in part, explains the variable clinical symptoms (Alberobello et al, 2011).

These examples illustrate well how the interplay between regulatory and coding sequences must be recognized as a fundamental feature of genome function, with important consequences for both phenotype and disease risk. In humans, this coupling helps explain variable penetrance and context-specific effects of disease mutations (Castel et al, 2018) (Fig. 3C). Genome-wide analyses, comparative studies, and in silico models have revealed widespread signatures of the effects of non-coding mutations in isolation. However, due to the lack of sufficient evidence from both clinical and genomics studies, it is unclear how coding mutations may modulate these effects of non-coding variants. Bridging this gap will be essential for translating the insights on the role of non-coding variation into informed predictive models of phenotype and disease.

At the same time, these findings reemphasize a broader evolutionary principle: mutations do not act in isolation, and their effects are shaped by the molecular and regulatory environments in which they occur. As seen across microbes, model organisms, and human populations, regulatory-coding epistasis constrains the paths available for evolution, shapes adaptive trajectories, and embeds molecular change within a broader genomic and cellular context. In the next section, we examine how this mode of epistasis reshapes our understanding of molecular evolution, affecting not just the outcomes of selection but the rules by which genetic variation is interpreted and acted upon.

## Implications for molecular evolution

Adaptive evolution can be conceptualized as a stepwise process in which beneficial mutations accumulate sequentially, each conferring a fitness advantage (Orr, 2002). Fitness landscapes provide a useful framework to capture genotype–phenotype relationships, and hence, adaptation. Empirical studies, mostly carried out using combinations of coding mutations in protein sequences, have revealed that fitness landscapes are rugged (Weinreich et al, 2006; Papkou et al, 2023; Starr et al, 2018; Sarkisyan et al, 2016). This ruggedness arises from underlying biophysical trade-offs, resulting in sign epistasis. For example, improving enzymatic efficiency may initially come at the cost of thermostability, which can later be restored through compensatory mutations (Knies et al, 2017; DePristo et al, 2005). On the other hand, a study that mapped the fitness landscape of expression using combinations of non-coding mutations found that pairs of beneficial mutations interacted non-epistatically at the level of gene expression, but antagonistically at the level of fitness. In these cases, the most beneficial single mutation often led to the most deleterious combinatorial outcome, a pattern explained by the nonlinear mapping from expression to fitness (as illustrated in Fig. 2C) (Chou et al, 2014; Chou and Marx, 2012).

**Figure 4:**
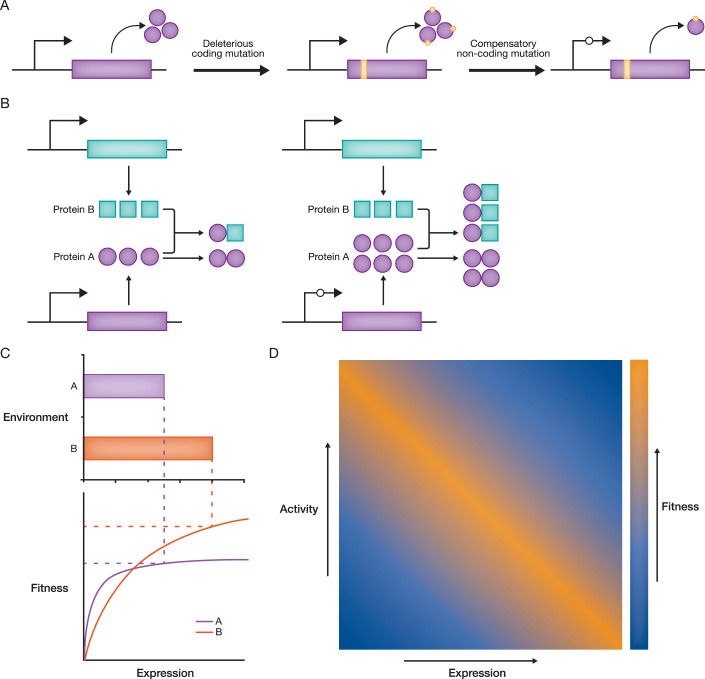
Implications of regulatory-coding epistasis on molecular evolution. (**A**) Consider a promoter–gene pair that encodes a protein not essential for cellular function. In such cases, the gene may accumulate mutations that are neutral in one context but deleterious in another. A promoter mutation that reduces expression can compensate for these deleterious effects by limiting the production of the deleterious protein variant. (**B**) Now consider two interacting proteins, A and B. Protein A forms both homodimers and heterodimers with protein B, and both complexes are essential for growth. When A is overexpressed due to a regulatory mutation, the levels of both homo- and heterodimers increase, potentially sequestering B. This imbalance can disrupt protein homeostasis and downstream metabolic processes. (**C**) Environmental conditions can influence the fitness effects of gene expression changes in two ways: either by directly altering expression levels alone (top panel) or by reshaping the fitness–expression relationship alone (bottom panel). The dashed lines connecting the two panels illustrate how these effects may combine, so that the same promoter can yield different fitness outcomes across environments depending on both expression changes and the mapping of expression to fitness. (**D**) Fitness, in this context, is a function of gene expression and activity of an essential protein. In this simplified schematic, we show that similar fitness can result from either low expression of a highly active protein or high expression of a less active one. The line connecting such points represents a “maximum fitness cline”; deviations from it reflect reduced fitness. The actual relationship may be nonlinear and context-dependent, varying with the protein and environment.

From the examples discussed above, it is clear that coding and regulatory changes rarely act independently on fitness. Together, they modulate the fitness effects of protein-coding mutations and impose additional constraints on adaptive trajectories. Yet, because much of their combinatorial mutational space remains unexplored, our understanding of the precise nature of constraints they impose is rudimentary. For instance, sign epistasis can arise even between two individually beneficial mutations, such as a regulatory change that increases gene expression and a coding change that enhances protein activity (Venkataraman and Saini, 2025). However, it is unclear whether the converse is possible: two individually deleterious mutations combining to produce a beneficial effect via regulatory-coding epistasis. The systematic deep-mutational scanning studies carried out to assay the expression-dependent fitness effects of single coding mutations reveal the molecular mechanisms that underlie the interdependence between coding and non-coding variants. While avoidance of translation errors has been proposed as the most important “constraint” in the evolution of coding sequences (Drummond and Wilke, 2008; Zhang and Yang, 2015), non-coding mutations may also mask the deleterious effects of coding mutations by altering their abundance and the consequent cytotoxicity (Fig. 4A) (Cisneros et al, 2023).

In addition to affecting the mutations that alter an existing function, expression levels can also shape the spectrum of coding mutations that enable the emergence of new functions. A directed evolution study in *E. coli* using GFP expressed at low and high levels showed that low expression facilitates the evolution of new phenotypes more effectively than high expression (Karve et al, 2022). This is because high expression, as shown by previously described examples, can buffer the deleterious effects of destabilizing mutations, allowing them to persist in the population. However, since protein stability promotes evolvability via the acquisition of destabilizing mutations (Tokuriki et al, 2008), populations that achieve a function through overexpression of unstable proteins are less likely to acquire neofunctionalizing variants than those that rely on optimal expression of well-folded proteins (Bloom et al, 2006; Tokuriki and Tawfik, 2009).

An additional layer of complexity in protein evolution arises from the genetic context in which mutations occur; specifically, whether gene variants are present in homoallelic or heteroallelic combinations in diploids, or paralogous copies in haploids (Domingo et al, 2019). The biophysical interactions between these gene products depend on the protein’s quaternary structure (e.g., homomeric vs. heteromeric assemblies) and the binding kinetics of individual subunits. These interactions influence the degree of dominance between alleles, which in turn affects organismal fitness (Pal et al, 2026). Importantly, allele-specific expression and mRNA levels, shaped by non-coding variation, can alter the stoichiometry of protein subunits and the composition of resulting complexes (Mallik et al, 2025; Badonyi and Marsh, 2023). Thus, non-coding mutations must be considered not only for their effects on expression levels but also for the plasticity they introduce in protein–protein interactions and their downstream fitness consequences (Fig. 4B) (Xie et al, 2023).

Finally, the phenotypic effects of mutations also depend on the environment, a phenomenon known as gene-by-environment (G×E) interactions. This arises because protein function, and consequently its role in cellular processes, can vary across environmental contexts. As a result, high expression of a nonessential gene is deleterious, but beneficial when the gene is essential (Wu et al, 2022). These shifts in fitness are driven by environment-dependent changes in both the genotype-to-expression (expression plasticity) and expression-to-fitness relationships (Siddiq et al, 2024). Studies at the interface of health and social sciences have revealed how changes in the social environment are correlated with alterations in gene expression profiles in humans, with important developmental and disease consequences (Heumann et al, 2025; Li et al, 2020; Vedhara et al, 2015; Apsley et al, 2024; Cole, 2014; Levine et al, 2017; Castagné et al, 2016; Guerrero et al, 2020; Ehrlich et al, 2015). Therefore, the interactions between coding and non-coding mutations are highly likely to vary with changes in the environment (Fig. 4C). Patterns from some high-throughput empirical studies discussed in this paper suggest that different combinations of protein abundance and activity can yield comparable fitness outcomes. For example, a highly efficient enzyme at low expression may confer the same fitness as a less efficient variant of the same enzyme expressed at its fitness-maximizing level, which depends on the cost of overexpression. The line connecting such points represents a maximum fitness cline, and deviations from it reflect a loss in fitness (Fig. 4D). In this simplified representation, the fitness landscape appears smooth rather than rugged, and indicates that several promoter–gene combinations may exist in a polymorphic population without any fitness consequences. The curvature and symmetry of the cline are unlikely to be as simple because they depend on the nonlinear contributions of activity and expression to fitness, as well as the physiological costs associated with modifying either trait. The shape of this cline plays a key role in shaping evolutionary outcomes. If the abundance–activity–fitness landscape is asymmetric, the distribution of fitness effects (DFE) of mutations will vary depending on the position of the wild-type enzyme along the cline. For instance, a poorly active enzyme that must be highly expressed to maintain function may be particularly sensitive to mutations that increase expression due to associated fitness costs. In contrast, for a highly efficient enzyme expressed at low levels, these costs may be negligible. As a result, both the mean and variance of the DFE will differ across genotypes, with consequences for evolvability, mutational robustness, and genetic load (Eyre-Walker and Keightley, 2007). While mutations affecting activity are well known to produce large fitness effects (Gerasimavicius et al, 2022), less is understood about the effect sizes of regulatory or expression-altering mutations (Kuo et al, 2020; Lagator et al, 2022). A recent work shows that only a small fraction of mutations, if any, in a yeast promoter provide adaptive benefits because of the nonlinear relationship between fitness and expression level (Aubé et al, 2025). However, it is unknown how coding mutations may interact with such a regulatory landscape.

Overall, regulatory-coding epistasis reshapes the adaptive landscape by modulating the distribution of fitness effects and altering the environment-dependence of mutational effects, that complicates the prediction of evolutionary trajectories. However, most evidence comes from a few studies, with limited exploration of the full combinatorial space in experimentally tractable contexts. In the following perspective, we consider what these findings imply for how we interpret genetic variation, predict evolutionary outcomes, and design future studies to uncover general principles of genome evolution, affecting not just the outcomes of selection but the rules by which genetic variation is interpreted and acted upon.

## Perspective

That coding and regulatory sequences coevolve and frequently interact epistatically challenges the view of molecular evolution as a linear accumulation of independent effects within each genomic region. Instead, what emerges is a highly interconnected, context-dependent genotype-to-phenotype map, where the effect of a single mutation depends not only on genetic changes that alter the biophysical properties of proteins but also on changes that modulate its expression, in an environment-dependent manner. This complexity significantly limits our ability to predict the phenotypic consequences of genetic variation across organisms, as well as our capacity to forecast the likelihood of adaptation or the structure of the underlying fitness landscape. Crucially, epistasis and its associated plasticity reveal that fitness landscapes are not fixed topographies but probabilistic surfaces or dynamic clouds of genotype–fitness relationships that shift with genetic and environmental context.

While our review focuses on the effects of individual adaptive mutations, natural populations often harbor multiple competing alleles, resulting in clonal interference. In such polymorphic populations, the fate of a mutation depends not only on its intrinsic effect but also on the presence of other beneficial alleles. Nonetheless, coarse-grained predictors, such as protein stability or background fitness, are likely to remain informative, shaping the direction and accessibility of evolutionary trajectories. Extending experimental approaches to polymorphic populations is an important future direction.

Because the fitness effect of any mutation depends on both background and environment, it becomes practically impossible to exhaustively assay all possible mutations across all genotypic and environmental combinations. This uncertainty underscores the need for new conceptual and experimental frameworks to map evolutionary potential in a holistic and predictive manner (Box 1).

High-throughput platforms such as multiplexed CRISPR perturbations, random mutagenesis, deep-mutational scanning with tunable promoters, or pooled variant libraries across expression gradients offer a promising path forward. Integrating biophysical models of protein folding and function, quantitative models of transcriptional regulation, and fitness landscapes measured across multiple environments with high-throughput mutagenesis will inform us of the mechanistic underpinnings of interactions between coding and non-coding regions, and potentially help generate macroscopic predictors of protein evolution. We can then apply this knowledge to interpret human genetic variation, engineer synthetic genomes, or understand the limits of adaptation.

Box 1 In need of answers

- How do cellular processes and constraints interact to shape the effects of mutations? A central challenge is to develop a generalizable framework for how expression level modulates the structure-function-fitness relationship. Current evidence indicates that proteins vary widely in their sensitivity to expression changes, but we lack a predictive theory that explains how fitness-abundance functions may change with mutations in the protein sequence. Clarifying how biophysical properties (e.g., folding landscapes, aggregation propensity, and interaction promiscuity) interact with physiological constraints (e.g., stoichiometric balance, flux control, compartmentalization) will be essential to predict when changes in expression amplify or buffer the effects of coding mutations.
- If the overexpression of some proteins is costlier than that of others, how do their evolutionary trajectories fundamentally differ? It may be predicted that dosage-sensitive proteins may be restricted to narrow and “fragile” fitness peaks, making both regulatory and coding mutations more constrained and likely favoring the evolution of tight feedback control. On the other hand, dosage-insensitive proteins may be able to accumulate greater regulatory variation, explore a wider mutational space, and contribute to adaption through diverse combinations of mutations. Understanding these differences empirically is key to predicting patterns of regulatory-coding evolution across genomes, especially in environments where expression demands fluctuate.
- How does protein abundance shape the emergence of neofunctionalizing proteins? Abundant proteins are exposed to far more molecular encounters per unit time, therefore have more chances to sample low-affinity or weakly beneficial activities. In principle, this elevated “encounter rate” could facilitate innovation: weak promiscuous activities become physiologically relevant at high concentrations, enabling the eventual evolution of new functions. We have discussed examples that suggest that high expression can temporarily compensate for low catalytic efficiency. However, high abundance, as we have detailed before, also imposes strong costs, and essential, highly expressed proteins are often under intense purifying selection. This dosage sensitivity may sharply limit the mutational space available for neofunctionalization. Thus, abundance may play a dual role: in nonessential, moderately expressed proteins, high expression can amplify weak promiscuous activities and accelerate functional divergence; but in essential or highly dosage-sensitive proteins, elevated abundance may suppress innovation by making most destabilizing or promiscuous mutations too costly to tolerate. Understanding how these opposing forces balance across protein classes is key to explaining why some proteins repeatedly give rise to new functions while others remain evolutionarily constrained.
- What is the role of gene expression noise in shaping regulatory-coding interactions? Many genes exhibit substantial cell-to-cell expression noise, yet most models of expression-fitness relationships rely on mean expression levels. For noisily expressed genes, fitness depends on the full distribution of expression across cells, not its average: rare cells with extremely low expression may limit population growth, while rare high-expression cells may pay disproportionate costs. This raises open questions about how organisms evolve to buffer or exploit noise, and how noise interacts with coding variation and causes epistasis at the level of fitness. For example, does expression noise increase the likelihood of regulatory-coding interactions by exposing coding mutations to a broader range of intracellular contexts?

## Supplementary information

Peer Review File
