# Supplementary material for: Molecular evolution in light of regulatory-coding epistasis
Source: EMBO Rep. 2026 Jun 10;27(13):3517–34. doi: 10.1038/s44319-026-00821-5 (PMC13354797; doi:10.1038/s44319-026-00821-5)
Supplement: Supplementary file 1 — Peer Review File [file 44319_2026_821_MOESM1_ESM.pdf]

EMBO reports

Molecular evolution in light of regulatory-coding epistasis

Pavithra Venkataraman and Christian Landry

Corresponding author(s): Pavithra Venkataraman (pavithra.venkataraman.1@ulaval.ca) , Christian Landry (christian.landry@bio.ulaval.ca)

Review Timeline:

Submission Date:	28th Aug 25
Editorial Decision:	17th Oct 25
Revision Received:	19th Dec 25
Editorial Decision:	2nd Apr 26
Revision Received:	8th Apr 26
Accepted:	29th Apr 26

Editor: Martina Rembold

Transaction Report:

(Note: With the exception of the correction of typographical or spelling errors that could be a source of ambiguity, letters and reports are not edited. Depending on transfer agreements, referee reports obtained elsewhere may or may not be included in this compilation. Referee reports are anonymous unless the Referee chooses to sign their reports.)

1st Editorial Decision 17th Oct 2025

Dear Christian,

Thank you for the submission of your revised review to EMBO reports. I apologize for this unusual delay in handling your manuscript but we have now received the full set of referee reports that is copied below.

As you will see, both referees consider the topic timely and interesting, but they also have a number of suggestions that I kindly ask you to take on board. You might want to expand the discussion on multicellular organisms and environmental effects that referee #1 comments on.

I think this is a very interesting review and while I appreciate that incorporating the referees' suggestions will still require a bit of work, I am convinced that the article is worth it and will benefit from it.

When submitting your revised manuscript, we will require a Microsoft Word file (.doc) of the revised manuscript text including detailed figure legends and the table, but without the figures. Please provide the figures as individual production quality files.

Please submit along with your review a detailed point-by-point response to the referee suggestions to facilitate the evaulation of your review.

I look forward to seeing a revised version of your manuscript when it is ready. Please let me know if you have questions or comments regarding the revision.

With best wishes,

Martina

Martina Rembold, PhD
Senior Editor
EMBO reports

====================

Referee #1:

This manuscript reviews current literature on genetic epistasis between coding and non-coding variation. The topic is interesting and the review is timely.
I was however disappointed in several respects. Many of them would be helped if the definition of aim and focus of the review was better framed at the very beginning and in the title, in part by narrowing down to specific adaptive evolutionary scenarios in specific organisms. Below are some remarks and suggestions.

- The review does not solely focus on unicellular organisms; however, the conceptual thinking is that of unicellular organisms. For example, it is never mentioned how important cis-regulatory variation may be regarding tissue specificity and pleiotropy in multicellular organisms. This is in my opinion a major issue.

- Environmental effects may be major when considering gene expression and are insufficiently taken in consideration. On a discussion on gene expression, I was expecting this in the first paragraph, at least on line 46. And examples thereafter.

- The whole angle of the manuscript is restricted to thinking about adaptive new mutations, not evolution in a polymorphic population with "alleles" rather than "mutations".

- All mapping is from genotype to fitness and it would be relevant to consider biological intermediates when explaining epistasis.

- Throughout, I see an ambiguity as to whether you refer to epistasis between coding and non-coding variation occurring within a gene (mostly or only?) or between two different genes.

- Figure 1A is a difficult explanation of epistasis (I got lost in the green lines). The scale of fitness is missing (log scale?). Orient graphs A and B similarly.
Panel C shows several molecular events leading to gene expression level, however the RNA level is fully missing (splicing, localization, stability, UTRs, etc.). The same remark applies for the text, for example on top of p. 7.

- Figure 2: the cost is not necessarily one in terms of energy or quality control machinery. Upregulation of, for example, a signaling molecule may be detrimental for other reasons. Again, the phenotypic consequences of gene expression levels are

missing.

- Figure 3 legend: the explanation of "distribution of fitness effects" is unclear. Overall, to conclude that "regulatory mutations can modulate both the expression and penetrance of disease-causing coding variants" is pretty obvious from the start. It is not epistasis as this sentence would also apply for additive effects.

- Whether the overall aim is to measure all possible epistatic interactions or to describe cases of actual evolution is ambiguous. The section on page 9 is particularly disappointing as most examples are from laboratory overexpression.

Writing:

- line 62: what is a "complete or comprehensive understanding of epistasis "?
- The structure at the end of the first paragraph and beginning of the second paragraph is weak. Idem lines 102-104.
- line 150 is unclear: what are the several orders of magnitude: different proteins or the same protein in different conditions, genotypes or cells?
- line 160: lysosomal pathways can be quite specific, for example through protein ubiquitinylation.
- lines 161-165; repeat. Why a focus on enhanced production?
- line 173: degradation may also dependent on the protein sequence. By focusing on some very specific examples of genetic variation, the richness of biological regulation is removed.
- lines 203-6: sentence structure
- line 454-5: rewrite

Referee #2:

>> Summary

Despite recognition that evolution arises from mutations in both regulatory elements and coding sequences, most prior studies have examined these sources of variation separately. By surveying work on metabolic systems, directed enzyme evolution, microbial evolution experiments, and genome-wide association studies of human traits, the authors demonstrate the functional dependence between coding and non-coding mutations, which can yield regulatory-coding epistasis of variable magnitude and even opposite sign. Such context dependence highlights a general evolutionary principle: mutations do not act in isolation but within molecular and regulatory environments that reshape their consequences.
The authors further discuss the implications of regulatory-coding epistasis and environment-dependent plasticity for altering the distribution of fitness effects, complicating efforts to predict adaptive trajectories. As a result, fitness landscapes are better conceived not as static topographies but as dynamic, probabilistic surfaces that shift with genetic and environmental context. To advance the field, the authors emphasize the need for new conceptual and experimental frameworks. They propose integrating genomics, synthetic biology, and biophysical modeling to capture the joint contributions of protein activity and abundance, with the ultimate goal of building predictive models for evolution, disease, and phenotypic diversity.

>> General comments

The review is well-written, constructive, and overall easy to follow. However, I cannot see a clear causal connection between "highly expressed genes evolve slowly" and "regulatory-coding epistasis" emphasized by the authors. Slow coding sequence evolution of highly expressed genes could be attributed to both strong purifying selection (gene essentiality) and the prevention of translational errors. The authors should either refine their argument or consider leaving this part out.
Also, some paragraphs are too short and better be merged together.

>> Detailed comments

L43-44. Cite relevant doi:
10.1038/s41580-018-0028-8,
10.1101/2025.01.23.634641,
and 210.1042/BST20120312

L44. "though" sounds awkward in this sentence.

L58-62. Cite the following examples in which epistasis occurs within (PMID: 32424071) and between non-coding regions (PMID: 21636771).

L94-96. Does this part mainly concern the effect of adaptive mutations occurring along the adaptive trajectories of microbial populations and does not concern deleterious mutations? This should be stated clearly. If this is about diminishing returns epistasis, the authors can cite PMID: 21636772, 21636771, 28812657.

L152-154. Shorten the phrase "...which may arise naturally in response to mutations, environmental perturbations, or be artificially engineered using synthetic biology tools,"

L169-170. Cite empirical evidence supporting this claim.

L191-197. The functional association between CNV of the ribosomal operon and ecological strategies of bacteria should be mentioned as well
doi: 10.1128/AEM.66.4.1328-1333.2000

L236-240. Cite doi: 10.1093/molbev/msm204

L358-361. "much higher" overclaims the impact of a single gene from a systems and control-theory perspective.

L454-455. grammar error "is reflects"

L459-464. Provide a brief physiological explanation for the contrasting fitness effect of dfrB1 mutations.

L469-487. I was a bit lost when reading this paragraph. To avoid that, the author should make the point "fitness effects of coding mutations depend not only on their biochemical properties but also on the level of gene expression" clear at the beginning of this paragraph. Also, the fact that highly expressed genes evolve more slowly likely attribute to both gene essentiality and avoiding translational/folding errors. Personally, I wouldn't use "challenge" here. I am also not sure if "highly expressed genes evolve more slowly" is a relevant example for the regulatory-coding epistasis, unlike the promoter-glutamate dehydrogenase dependence.

L553. "core" promoter region or promoter proximal element?

L562-565. Briefly explain the basis (statistical/functional) of this study's interpretation.

L565-568. Similar to my question about "highly expressed genes evolve more slowly", is the observation here due to gene essentiality or regulatory-coding epistasis?

L622-624. The modes of action of these non-coding mutations are described in PMID: 22832162. Cite this reference for a mechanistic explanation.

L701-704. Cite two relevant DMS examples for expression-altering mutations doi:
10.1101/gr.260182.119
10.7554/eLife.64543

Fig. 1A. The stacked histogram can be plotted in a more intuitive manner. Particularly, I found using diagonal -line boxes to indicate ranges perplexing. I had to read the legend twice to get the meaning of fn and fs.

Fig 1C should include the post-transcriptional regulation step as it is particularly important for eukaryotic gene expression.

Fig. 2C. The magnitude of the fitness burden is too large.

Response to reviewer comments:

We thank both reviewers for their feedback and specific comments. The manuscript has been edited based on the comments, and the edited text is highlighted in green. The specific responses to each of the comments can be found below. We have also pasted the edited text/figures from the manuscript for the convenience of the reviewers and the editor.

Referee #1:

This manuscript reviews current literature on genetic epistasis between coding and non-coding variation. The topic is interesting and the review is timely.

I was however disappointed in several respects. Many of them would be helped if the definition of aim and focus of the review was better framed at the very beginning and in the title, in part by narrowing down to specific adaptive evolutionary scenarios in specific organisms. Below are some remarks and suggestions.

- The review does not solely focus on unicellular organisms; however, the conceptual thinking is that of unicellular organisms. For example, it is never mentioned how important cis-regulatory variation may be regarding tissue specificity and pleiotropy in multicellular organisms. This is in my opinion a major issue.

Response: This has been considered, and we did this on purpose to narrow down on the best known examples. We now make this choice clear with several edits to indicate that we are drawing lessons from studies on unicellular organisms and extending them to predict what would happen in more complex organisms.

protein abundance and activity jointly shape fitness, constrain adaptation, and impact molecular evolution. Drawing on examples from systematic studies carried out in unicellular organisms, we speculate on a fitness function integrating abundance and activity and discuss the broader implications of these interactions for evolutionary dynamics, genetic disease, and phenotypic diversity.

protein abundance and function (Buchberger *et al*, 2019; Katsonis *et al*, 2014). Tracking the adaptive evolutionary impact of mutations in non-coding regions is challenging, in part because they are highly variable across taxa - in eukaryotes, non-coding regions are vast, but much smaller in bacteria and archaea (Haberle & Stark, 2018; Kuo *et al*, 2025; Peeters *et al*, 2013). In most cases, they lack well-defined functional genomic annotations (Guigó, 2023). The effects of non-coding changes are often genetic context-dependent (Brown *et al*, 2009; McQueen *et al*, 2025), environment-dependent (Featherstone & Broadie, 2002; Vande Zande & Wittkopp, 2022; Wittkopp *et al*, 2008; McManus *et al*, 2010), and in some cases in multicellular organisms, cell-dependent (Scacheri & Scacheri, 2015). Therefore, their effects are difficult to

Epistatic relationships are additionally complicated by genotype-by-environment (GxE) interactions, which modulate the fitness outcomes of mutations, largely based on the need of the protein they affect (Johannsen, 1911). The environment can act at two levels to modify the fitness of genotypes (Aubé & Landry, 2024). It can alter the mapping between mutations and activity and function of the genes and between activity and function and fitness. Generally, changing environments could alter regulation dynamics (Penner-Goeke & Binder, 2024), protein abundance (Munro *et al*, 2024), metabolic state of the cell (Mallard *et al*, 2018) and modify where the optimality of protein activity is (Dekel & Alon, 2005). Understanding protein evolution, therefore, requires an integrated approach that accounts for the regulatory architecture governing gene expression, the structural and functional impacts of coding mutations, and the influence of the genomic and environmental context on their interplay (**Fig 1C**).

- Environmental effects may be major when considering gene expression and are insufficiently taken in consideration. On a discussion on gene expression, I was expecting this in the first paragraph, at least on line 46. And examples thereafter.

Response: We have expanded our discussion on the role of the environment:

Fig 1. The fitness effects of non-coding mutations depend on the genetic background and the environment. (A) This cartoon illustrates how the fitness effect of a mutation (a→A in a specific site) changes across two genetic backgrounds: *b* (solid green line) and *B* (dashed pink line). In the no epistasis case, the fitness effect of the mutation is identical in both backgrounds. In the positive epistasis case, the mutation's effect is larger in background *B* than in *b*. But when there is negative epistasis, the mutation still increases fitness, but its effect is smaller in *B* than in *b*. Sign epistasis represents an extreme scenario in which the background changes the direction of effect itself: the mutation is beneficial in background *b* but not in *B.* In general, sign epistasis produces a fitness effect whose magnitude is smaller than that observed in no epistasis condition. (B) Examples of epistasis between pairs of mutations are well documented. Here, we illustrate a case of epistasis between a regulatory and a coding mutation (inspired by Brown *et. al.*) (Brown *et al*, 2009). The wild-type genotype has the lowest fitness. A regulatory mutation (blue square) alone provides only a small fitness benefit, while a coding mutation (orange triangle) alone leads to a larger increase in fitness. However, when the regulatory mutation occurs on the background of the coding mutation, its fitness effect is much greater than when it occurs on the wild-type background. This suggests a form of positive epistasis, where the regulatory mutation is more beneficial in the presence of the coding change. In an adaptive context, this implies that the coding mutation is likely fixed first, followed by the regulatory mutation. (C) While the fitness effects of mutations depend on both the genetic background and the environment, the underlying causes often involve one or more molecular changes. Genomic and environmental alterations can affect the abundance and composition of RNA polymerases, ribosomes, amino acids, and the proteome, including post-transcriptional and post-translational regulators depending on the organism's complexity, protein degradation machinery, and other interacting proteins, as well as how the cell imports chemicals from its surroundings. Together, these changes influence promoter activity, transcription, translation, protein abundance, protein–protein interactions, and ultimately, cellular function and fitness.

Epistatic relationships are additionally complicated by genotype-by-environment (GxE) interactions, which modulate the fitness outcomes of mutations, largely based on the need of the protein they affect (Johannsen, 1911). The environment can act at two levels to modify the fitness of genotypes (Aubé & Landry, 2024). It can alter the mapping between mutations and activity and function of the genes and between activity and function and fitness. Generally, changing environments could alter regulation dynamics (Penner-Goeke & Binder, 2024), protein abundance (Munro *et al*, 2024), metabolic state of the cell (Mallard *et al*, 2018) and modify where the optimality of protein activity is (Dekel & Alon, 2005). Understanding protein evolution, therefore, requires an integrated approach that accounts for the regulatory architecture governing gene expression, the structural and functional impacts of coding mutations, and the influence of the genomic and environmental context on their interplay (**Fig 1C**).

- The whole angle of the manuscript is restricted to thinking about adaptive new mutations, not evolution in a polymorphic population with "alleles" rather than "mutations".

Response: Thanks for the useful comment. The manuscript has been edited to add this perspective to our review.

fitness cline, and deviations from it reflect a loss in fitness (**Fig 4D**). In this simplified representation, the fitness landscape appears smooth rather than rugged, and indicates that several promoter-gene combinations may exist in a polymorphic population without any fitness consequences. The curvature and symmetry of the cline are unlikely to be as simple because

While our review focuses on the effects of individual adaptive mutations, natural populations often harbor multiple competing alleles, resulting in clonal interference. In such polymorphic populations, the fate of a mutation depends not only on its intrinsic effect but also on the presence of other beneficial alleles. Nonetheless, coarse-grained predictors, such as protein stability or background fitness, are likely to remain informative, shaping the direction and accessibility of evolutionary trajectories. Extending experimental approaches to polymorphic populations is an important future direction.

- All mapping is from genotype to fitness and it would be relevant to consider biological intermediates when explaining epistasis.

Response: While this is true, in most experimental studies, fitness is the measure of mutational effects and is thought to be most relevant for adaptation. The manuscript has been edited to add this clarity. Wherever possible, we have included information about biological intermediates.

In this review, we examine how variation in protein abundance, together with changes in structure and activity, shapes the adaptive evolutionary dynamics of proteins by constraining or facilitating adaptation. In this Darwinian context, fitness serves as the most relevant measure of evolutionary success, even though genotype–phenotype relationships are mediated by molecular and physiological intermediates that are often poorly characterized and/or understood. Drawing on evidence from several organisms, we derive insights that inform our understanding of genetic disease, phenotypic diversity, and general principles of molecular evolution.

- Throughout, I see an ambiguity as to whether you refer to epistasis between coding and non-coding variation occurring within a gene (mostly or only?) or between two different genes.

Response: Thanks, we have revised the text to clarify that our primary focus is on regulatory–coding epistasis, where cis-regulatory variants affect the gene's expression and coding variants affect protein function or cost, and where the most detailed empirical examples currently exist. We also note that regulatory–coding epistasis can occur between different genes, particularly in complex organisms, and we now make this distinction explicit where relevant.

- Figure 1A is a difficult explanation of epistasis (I got lost in the green lines). The scale of fitness is missing (log scale?). Orient graphs A and B similarly.

Panel C shows several molecular events leading to gene expression level, however the RNA level is fully missing (splicing, localization, stability, UTRs, etc.). The same remark applies for the text, for example on top of p. 7.

Response: Fig. 1A has been altered to explain epistasis more simply. The caption has been edited to explain that Fig. 1C is not all-encompassing, but only a representation of the complexity of molecular processes.

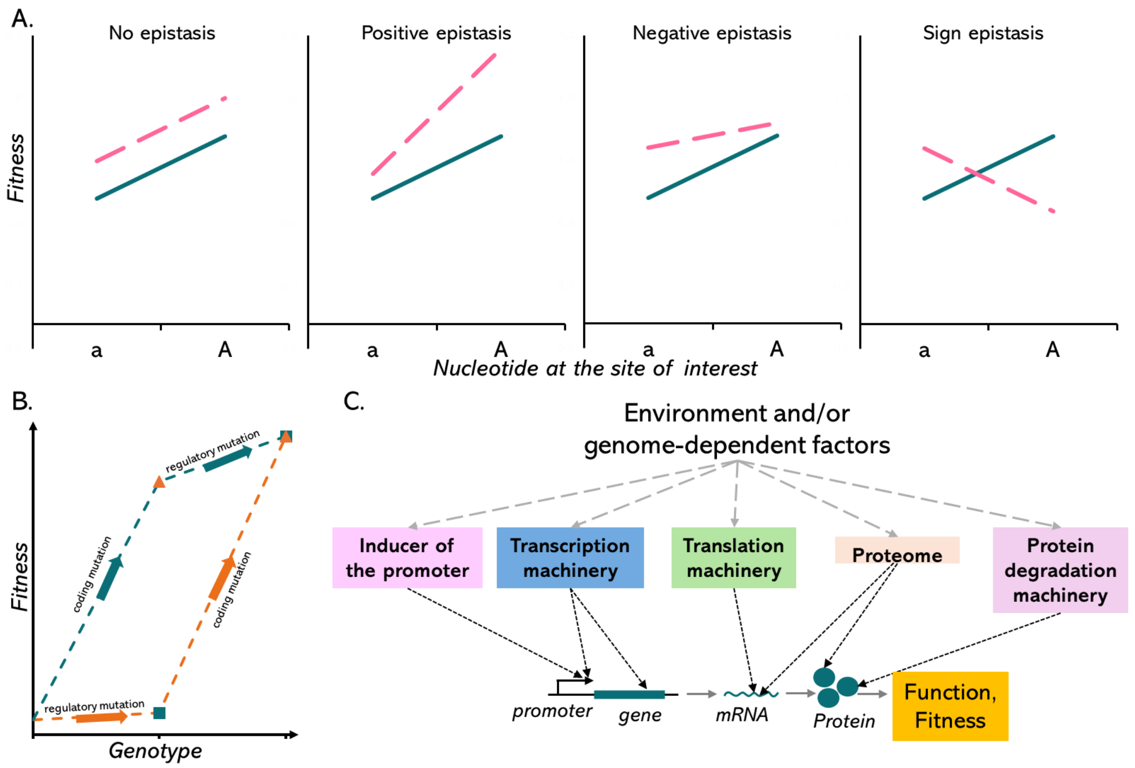

Fig 1. The fitness effects of non-coding mutations depend on the genetic background and the environment. (A) This cartoon illustrates how the fitness effect of a mutation (a→A in a specific site) changes across two genetic backgrounds: *b* (solid green line) and *B* (dashed pink line). In the no epistasis case, the fitness effect of the mutation is identical in both backgrounds. In the positive epistasis case, the mutation's effect is larger in background *B* than in *b*. But when there is negative epistasis, the mutation still increases fitness, but its effect is smaller in *B* than in *b*. Sign epistasis represents an extreme scenario in which the background changes the direction of effect itself: the mutation is beneficial in background *b* but not in *B*. In general, sign epistasis produces a fitness effect whose magnitude is smaller than that observed in no epistasis condition. (B) Examples of epistasis between pairs of mutations are well documented. Here, we illustrate a case of epistasis between a regulatory and a coding mutation (inspired by Brown *et. al.*) (Brown *et al*, 2009). The wild-type genotype has the lowest fitness. A regulatory mutation (blue square) alone provides only a small fitness benefit, while a coding mutation (orange triangle) alone leads to a larger increase in fitness. However, when the regulatory mutation occurs on the background of the coding mutation, its fitness effect is much greater than when it occurs on the wild-type background. This suggests a form of positive epistasis, where the regulatory mutation is more beneficial in the presence of the coding change. In an adaptive context, this implies that the coding mutation is likely fixed first, followed by the regulatory mutation. (C) While the fitness effects of mutations depend on both the genetic background and the environment, the underlying causes often involve one or more molecular changes. Genomic and environmental alterations can affect the abundance and composition of RNA polymerases, ribosomes, amino acids, and the proteome, including post-transcriptional and post-translational regulators depending on the organism's complexity, protein degradation machinery, and other interacting proteins, as well as how the cell imports chemicals from its surroundings. Together, these changes influence promoter activity, transcription, translation, protein abundance, protein–protein interactions, and ultimately, cellular function and fitness.

- Figure 2: the cost is not necessarily one in terms of energy or quality control machinery. Upregulation of, for example, a signaling molecule may be detrimental for other reasons. Again, the phenotypic consequences of gene expression levels are missing.

Response: Thanks for the comment. The cost of overexpression can stem from a variety of reasons, including the upregulation of a signaling molecule. The fitness effects of such costs have been empirically measured previously, and the new figure incorporates these findings. At the molecular level, overexpression of any molecule eventually overwhelms the quality control/stress response machinery.

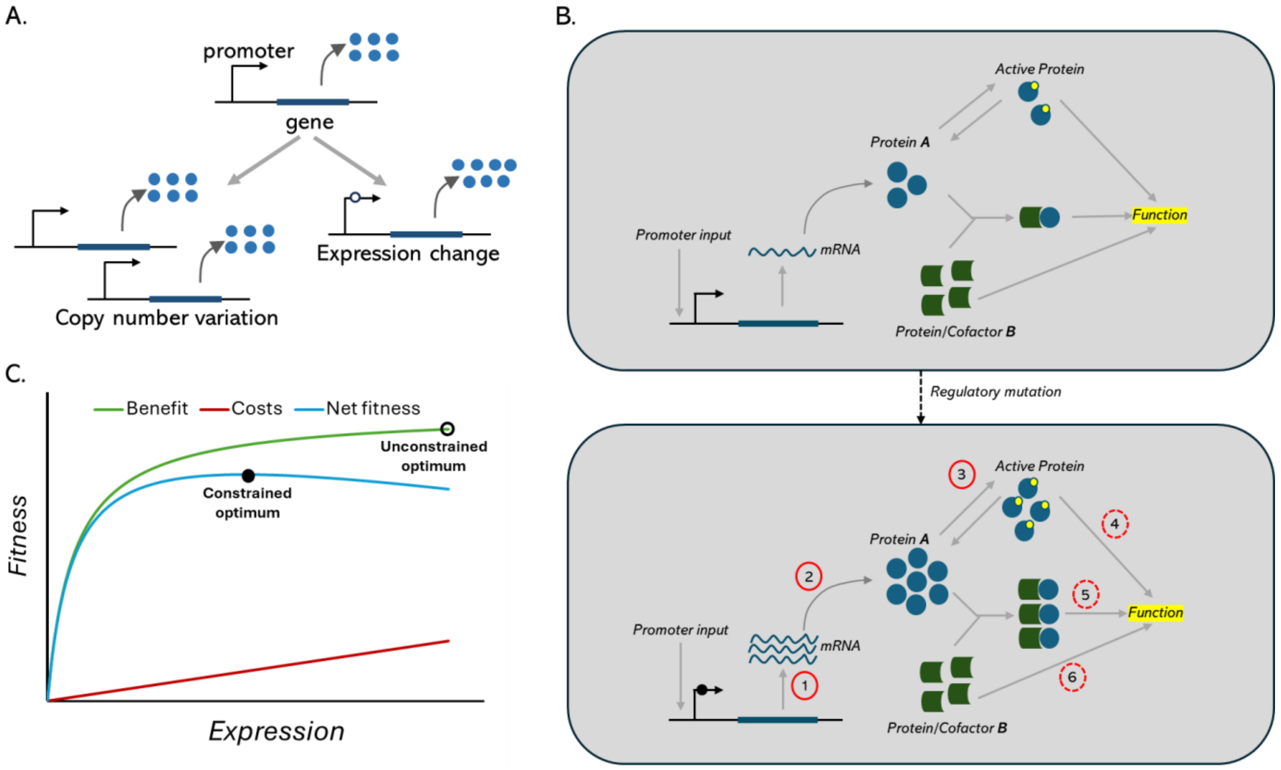

- Figure 3 legend: the explanation of "distribution of fitness effects" is unclear. Overall, to conclude that "regulatory mutations can modulate both the expression and penetrance of disease-causing coding variants" is pretty obvious from the start. It is not epistasis as this sentence would also apply for additive effects.

Response: Thanks. The caption has been edited to make the definition of DFE clear, and to explain how regulatory-coding epistasis may affect penetrance.

Fig 3. Regulatory-coding epistasis and its effect on disease risk. (A) The fitness effects of a library of single amino acid mutations in yeast Hsp90 were measured across different expression levels. The figure (based on the average fitness effect data from Jiang *et. al.* *(Jiang et al, 2013)*) shows the distribution of fitness effects (DFE), which describes the range and likelihood of fitness consequences of new mutations (*s*). The DFE clearly shifts with expression: higher expression levels, by increasing transcription, buffer the deleterious effects of some mutations but also reduce the benefits of others, resulting in a narrower distribution. (B) A similar, more comprehensive deep mutational scanning study of the *dfrB1* gene in *E. coli* further highlights the influence of expression on mutational effects. The figure (based on the average fitness effect data from Cisneros *et. al.* (Cisneros *et al*, 2023)) compares fitness effects at high or intermediate expression levels (promoter activity) with fitness at low expression. Increased expression buffers mildly deleterious mutations but also reveals the costs of overexpression, as beneficial mutations show reduced fitness gains at higher expression levels. (C) This example (inspired by Castel *et. al.* (Castel *et al*, 2018)) illustrates how regulatory and coding mutations can interact to produce a disease phenotype in a heterogeneous population. In the wild-type state, the gene is not transcribed, and no protein is produced, resulting in the lowest disease penetrance. A regulatory mutation (solid blue circle with black border) can activate transcription in the homozygous state, leading to protein production (solid blue circles) and increased penetrance, but comparable to that caused by a homozygous deleterious coding mutation (yellow four-point stars) that is not transcribed. However, when individuals are homozygous for both the regulatory and the coding mutations, the expressed mutant protein (blue circles with red borders) causes a disease phenotype in most individuals. Thus, regulatory mutations can non additively modulate both the penetrance of disease-causing coding variants.

- Whether the overall aim is to measure all possible epistatic interactions or to describe cases of actual evolution is ambiguous.

Response: It was unclear to us which section was unclear but we hope that it has been corrected with the many changes we have made.

The section on page 9 is particularly disappointing as most examples are from laboratory overexpression.

Response: Thanks for the comment. While the section starts with a description of overexpression screens, there are several examples of overexpression reported from evolution experiments in plants and mice. In the section on human genetics, we have more examples that are seen in humans and that are not from lab studies.

factor binding sites (Chen *et al*, 2023). In the plant *Arabidopsis thaliana*, overexpression of a GTP-binding protein enhanced resistance to the pathogen *Pseudomonas syringae* DC3000, widely known for causing disease in tomato plants (Xu *et al*, 2021). In mice, the overexpression of STIL, a gene involved in cell cycle regulation, reduces the likelihood of tumor formation, but at the cost of reduced lifespan, indicating that overexpression may have constraints that result in fitness “costs” (Moussa *et al*, 2023).

In 1975, King and Wilson proposed that most of the morphological differences between humans and our closest relative, the chimpanzee, stem from changes in non-coding regions, rather than coding ones (King & Wilson, 1975). Their work using blood proteins was one of the earliest to demonstrate the importance of studying the evolution of regulatory mechanisms in humans, as protein sequences appear to evolve too slowly to explain human specificity. Non-coding mutations in the human genome, like in other organisms, contribute to phenotypes by altering gene expression through the modulation of transcription factor binding, alterations to gene expression patterns, and post-translational modifications (Peña-Martínez & Rodríguez-Martínez, 2024; Stranger & Dermitzakis, 2005; Cheng *et al*, 2024; van der Lee *et al*, 2020). An early example of the role of regulatory mutations in disease phenotypes comes from a 1982 study, which reported that single-nucleotide polymorphisms (SNPs) in the promoter of the β-globin gene were linked to β-thalassemia (Orkin *et al*, 1982). Since then, and in the post-genomics era, it has been challenging to determine which of the millions of mutations in the huge non-coding regions of the human genome are responsible for phenotypes. The Encyclopaedia of DNA Elements (ENCODE) consortium was established in 2004 to address this challenge. The initial findings of this project suggested that about 80% of the genome has some biochemical activity, and previously understudied regions such as enhancers, silencers, and untranslated regions play regulatory roles (ENCODE Project Consortium, 2004). While subsequent analyses have revised the estimated functional fraction of the genome downward (Graur, 2017; Christmas *et al*, 2023), genome-wide association studies have revealed that over 90% of disease- and phenotype-associated variation originates from non-coding regions (Peña-Martínez & Rodríguez-Martínez, 2024; Lee *et al*, 2018; Maurano *et al*, 2012; Buniello *et al*, 2019; Deplancke *et al*, 2016; Zhang & Lupski, 2015). Recent estimates indicate that 90% of mutations in enhancers are neutral, and approximately 30% of mutations in core promoter regions are deleterious (Di *et al*, 2025).

Writing:

- line 62: what is a "complete or comprehensive understanding of epistasis "?

Response: The line has been edited.

combinations of mutations often unpredictable from the effect of single ones (Sandhu *et al*, 2025). Therefore, in the absence of a mechanistic understanding of how epistasis operates at the cellular level, predicting evolution requires that the fitness effect of every mutation in every possible genotype is measured in as many environments as possible. However, as the genome

- The structure at the end of the first paragraph and beginning of the second paragraph is weak. Idem lines 102-104.

Response: The section has been edited.

Colunga *et al*, 2023). Background fitness thus acts as a macroscopic predictor of evolutionary outcomes.

A natural question is whether an analogous, coarse-grained predictor exists at the level of individual proteins. One prominent candidate is protein stability. A growing body of work

- line 150 is unclear: what are the several orders of magnitude: different proteins or the same protein in different conditions, genotypes or cells?

Response: The line has been edited.

The abundance of individual proteins in a cell can differ by several orders of magnitude (Munro *et al*, 2024). Yet, even small changes in abundance can significantly impact phenotypes across

- line 160: lysosomal pathways can be quite specific, for example through protein ubiquitinylation.

Response: The section has been edited.

Protein levels can be modified by altering either their production, or consumption. Although proteins are not “consumed” in the classical metabolic sense, they transition between conformational states (Bryan & Orban, 2010), and are degraded through pathways that maintain cellular homeostasis. Degradation can be non-specific, as in some lysosomal pathways, or targeted, as in the ubiquitin-proteasome system (Zhao *et al*, 2022; Cooper, 2000). Mutations in these pathways can therefore cause global, or substrate-specific shifts in protein abundance, depending on which components are perturbed (Chiba & Tanaka, 2005). In yeast, for example, several polymorphisms modulate the activity of the ubiquitin-proteasome protein degradation system, often in substrate-specific ways (Collins *et al*, 2022). Although we know considerably less about how genetic variation affects degradation rates quantitatively, recent work shows that degradation itself is environment-dependent (Avery *et al*, 2025). In rapidly growing cells, however, dilution by cell division is often considered to be a major contributor to decreases in abundance of several classes of proteins (Gupta *et al*, 2024).

- lines 161-165; repeat. Why a focus on enhanced production?

Response: The section has been removed.

- line 173: degradation may also dependent on the protein sequence. By focusing on some very specific examples of genetic variation, the richness of biological regulation is removed.

Response: The reviewer is right that protein degradation rates can depend on the protein sequence. In the revision, we now explicitly discuss genetic and environmental variation in degradation pathways and cite recent work demonstrating that degradation rates are themselves environment and genetic background-dependent. While our primary focus remains on regulatory–coding interactions affecting protein production, these same principles extend to degradation, which can also mediate epistasis between coding changes and regulatory context when degradation rates are modified by mutations.

Protein levels can be modified by altering either their production, or consumption. Although proteins are not “consumed” in the classical metabolic sense, they transition between conformational states (Bryan & Orban, 2010), and are degraded through pathways that maintain cellular homeostasis. Degradation can be non-specific, as in some lysosomal pathways, or targeted, as in the ubiquitin-proteasome system (Zhao *et al*, 2022; Cooper, 2000). Mutations in these pathways can therefore cause global, or substrate-specific shifts in protein abundance, depending on which components are perturbed (Chiba & Tanaka, 2005). In yeast, for example, several polymorphisms modulate the activity of the ubiquitin-proteasome protein degradation system, often in substrate-specific ways (Collins *et al*, 2022). Although we know considerably less about how genetic variation affects degradation rates quantitatively, recent work shows that degradation itself is environment-dependent (Avery *et al*, 2025). In rapidly growing cells, however, dilution by cell division is often considered to be a major contributor to decreases in abundance of several classes of proteins (Gupta *et al*, 2024).

In contrast, protein production is governed by transcriptional and translational control, which are directly shaped by both regulatory (non-coding) and coding sequences. Common mechanisms to alter production include changes in gene copy number, transcriptional activity, and translation rates (Kafri *et al*, 2016). These processes are comparatively well understood, and consequently, most of our knowledge about how genetic variation affects abundance and activity comes from changes in production rather than degradation.

Nevertheless, the same conceptual principles apply to any regulatory step whose rate can be altered by mutations. This framework underlies regulatory-coding epistasis, in which non-coding mutations affect expression and coding mutations alter protein activity, stability, or abundance, and the combined effects interact non-additively to determine phenotypes.

- lines 203-6: sentence structure

Response: The line has been edited.

In humans, one of the most noted examples of adaptation via CNVs comes from the genes that code for α-amylase enzymes. All three genes, AMY1 (salivary), AMY2A, and AMY2B (pancreatic), show variable copy numbers across individuals that correlate with the amount of starch in the diet of the populations (Groot *et al*, 1989, 1990). While the reference genome (GRCh38) contains three copies of AMY1, copy numbers can range widely, especially for AMY1. In contrast, other great apes lack such variation, typically carrying a single copy of each gene. These genes arose through ancestral duplications, but recurrent CNVs are unique to modern humans, absent in Neanderthals and Denisovans (Inchley *et al*, 2016). This makes the amylase locus a curious case of rapid structural evolution, likely shaped by dietary shifts (Pajic *et al*, 2019).

- line 454-5: rewrite

Response: The line has been edited.

library of coding mutations with different expression strengths to elucidate the evolutionary constraints on coding and non-coding sequences. In these contexts, modifying expression levels reflects the potential effects of mutations on expression levels. For example, in a mutational

Referee #2:

>> Summary

Despite recognition that evolution arises from mutations in both regulatory elements and coding sequences, most prior studies have examined these sources of variation separately. By surveying work on metabolic systems, directed enzyme evolution, microbial evolution experiments, and genome-wide association studies of human traits, the authors demonstrate the functional dependence between coding and non-coding mutations, which can yield regulatory-coding epistasis of variable magnitude and even opposite sign. Such context dependence highlights a general evolutionary principle: mutations do not act in isolation but within molecular and regulatory environments that reshape their consequences.

The authors further discuss the implications of regulatory-coding epistasis and environment-dependent plasticity for altering the distribution of fitness effects, complicating efforts to predict adaptive trajectories. As a result, fitness landscapes are better conceived not as static topographies but as dynamic, probabilistic surfaces that shift with genetic and environmental context. To advance the field, the authors emphasize the need for new conceptual and experimental frameworks. They propose integrating genomics, synthetic biology, and biophysical modeling to capture the joint contributions of protein activity and abundance, with the ultimate goal of building predictive models for evolution, disease, and phenotypic diversity.

>> General comments

The review is well-written, constructive, and overall easy to follow. However, I cannot see a clear causal connection between "highly expressed genes evolve slowly" and "regulatory-coding epistasis" emphasized by the authors. Slow coding sequence evolution of highly expressed genes could be attributed to both strong purifying selection (gene essentiality) and the prevention of translational errors. The authors should either refine their argument or consider leaving this part out.

Response: Thanks for this comment. The reviewer is right in pointing out slow coding sequence evolution of highly expressed genes may be due to purifying selection or prevention of translational errors. However, regulatory-coding epistasis is a fitness-level phenomenon that results from several molecular causes, including the ones that drive the slow evolution of overexpressed genes.

Also, some paragraphs are too short and better be merged together.

Response: Short paragraphs have been merged throughout the manuscript.

>> Detailed comments

L43-44. Cite relevant doi:

10.1038/s41580-018-0028-8,

10.1101/2025.01.23.634641,

and 210.1042/BST20120312

Response: Thanks, these references have been added.

protein abundance and function (Buchberger *et al*, 2019; Katsonis *et al*, 2014). Tracking the adaptive evolutionary impact of mutations in non-coding regions is challenging, in part because they are highly variable across taxa - in eukaryotes, non-coding regions are vast, but much smaller in bacteria and archaea (Haberle & Stark, 2018; Kuo *et al*, 2025; Peeters *et al*, 2013). In

L44. "though" sounds awkward in this sentence.

Response: The line has been edited.

protein abundance and function (Buchberger *et al*, 2019; Katsonis *et al*, 2014). Tracking the adaptive evolutionary impact of mutations in non-coding regions is challenging, in part because they are highly variable across taxa - in eukaryotes, non-coding regions are vast, but much smaller in bacteria and archaea (Haberle & Stark, 2018; Kuo *et al*, 2025; Peeters *et al*, 2013). In

L58-62. Cite the following examples in which epistasis occurs within (PMID: 32424071) and between non-coding regions (PMID: 21636771).

Response: Thanks, these references have been added.

genetic loci, is pervasive (Bateson & Mendel, 1909). Epistasis can occur within a single coding region (Starr & Thornton, 2016), between distinct coding loci (Johnson *et al*, 2023), within non-coding regions (Kuo *et al*, 2020; Ang *et al*, 2023; Bernet & Elena, 2015), between non-coding regions (McQueen *et al*, 2025), or across regulatory and coding elements (Lagator *et al*, 2017). Any of these forms of epistasis alters mutational effects in non-additive ways (**Fig**

1A), making the fitness effects of combinations of mutations often unpredictable from the effect of single ones (Sandhu *et al*, 2025). Therefore, in the absence of a mechanistic understanding of how epistasis operates at the cellular level, predicting evolution requires that

L94-96. Does this part mainly concern the effect of adaptive mutations occurring along the adaptive trajectories of microbial populations and does not concern deleterious mutations? This should be stated clearly. If this is about diminishing returns epistasis, the authors can cite PMID: 21636772, 21636771, 28812657.

Response: Thanks, these references have been added.

Predictions of fitness changes in microbial evolution experiments have been made through seemingly simple principles. Over the past decade, evolution experiments have shown that the average fitness effect of an adaptive mutation is typically smaller in high-fitness backgrounds than in low-fitness backgrounds, indicating negative epistasis between beneficial mutations in an evolving population, resulting in temporally diminishing-returns at the level of fitness (Khan *et al*, 2011; Chou *et al*, 2011; Wünsche *et al*, 2017). This means that, given the fitness effect of a mutation in one background, its effect in other backgrounds can often be predicted without

L152-154. Shorten the phrase "...which may arise naturally in response to mutations, environmental perturbations, or be artificially engineered using synthetic biology tools,"

Response: The lines have been modified accordingly.

The abundance of individual proteins in a cell can differ by several orders of magnitude (Munro *et al*, 2024). Yet, even small changes in abundance can significantly impact phenotypes across organisms, from microbes to humans. Such changes, whether environment-induced, caused by spontaneous mutations or synthetically engineered, shape both survival and long-term evolution.

L169-170. Cite empirical evidence supporting this claim.

Response: The whole section has been altered.

Protein levels can be modified by altering either their production, or consumption. Although proteins are not “consumed” in the classical metabolic sense, they transition between conformational states (Bryan & Orban, 2010), and are degraded through pathways that maintain cellular homeostasis. Degradation can be non-specific, as in some lysosomal pathways, or targeted, as in the ubiquitin-proteasome system (Zhao *et al*, 2022; Cooper, 2000). Mutations in these pathways can therefore cause global, or substrate-specific shifts in protein abundance, depending on which components are perturbed (Chiba & Tanaka, 2005). In yeast, for example, several polymorphisms modulate the activity of the ubiquitin-proteasome protein degradation system, often in substrate-specific ways (Collins *et al*, 2022). Although we know considerably less about how genetic variation affects degradation rates quantitatively, recent work shows that degradation itself is environment-dependent (Avery *et al*, 2025). In rapidly growing cells, however, dilution by cell division is often considered to be a major contributor to decreases in abundance of several classes of proteins (Gupta *et al*, 2024).

In contrast, protein production is governed by transcriptional and translational control, which are directly shaped by both regulatory (non-coding) and coding sequences. Common mechanisms to alter production include changes in gene copy number, transcriptional activity, and translation rates (Kafri *et al*, 2016). These processes are comparatively well understood, and consequently, most of our knowledge about how genetic variation affects abundance and activity comes from changes in production rather than degradation.

Nevertheless, the same conceptual principles apply to any regulatory step whose rate can be altered by mutations. This framework underlies regulatory-coding epistasis, in which non-coding mutations affect expression and coding mutations alter protein activity, stability, or abundance, and the combined effects interact non-additively to determine phenotypes.

L191-197. The functional association between CNV of the ribosomal operon and ecological strategies of bacteria should be mentioned as well
doi: 10.1128/AEM.66.4.1328-1333.2000

Response: Thanks, these references have been added.

[illegible] associated with CNVs is stress response. For instance, in plants, CNVs have been shown to confer herbicide resistance (Gaines *et al*, 2010). In microbes, where evolutionary and molecular mechanisms are often studied in detail, CNVs underlie resistance to chemicals (von Rozycki & Nies, 2009; Chow *et al*, 2012; Adamo *et al*, 2012; Fijarczyk *et al*, 2020) and antibiotics (Sandegren & Andersson, 2009; Craven & Neidle, 2007; Price *et al*, 2004; Segovia, 1994; Mary *et al*, 2010; Soo *et al*, 2011; Selmecki *et al*, 2006; Sionov *et al*, 2010; Ni *et al*, 2013; Berman & Krysan, 2020). CNVs also contribute to adaptation in nutrient-limiting (Harari *et al*, 2018; Gresham *et al*, 2008, 2010; Brown *et al*, 1998), osmotic (Dhar *et al*, 2011), thermal stress conditions (Riehle *et al*, 2001; Christ & Chin, 2008), and are thought to contribute to variation in ecological strategies (Klappenbach *et al*, 2000; Raj & Saini, 2024). In most of these cases, fitness benefit is attributed to the increased protein dosage.

L236-240. Cite doi: 10.1093/molbev/msm204

Response: Thanks, this reference has been added.

whereby a more abundant drug target is expected to lead to resistance to the drug. Additionally, overexpression screens have also helped identify non-cognate *E. coli* genes which when upregulated, rescue cells with metabolic auxotrophies (Patrick *et al*, 2007). On the other hand, evolution experiments revealed candidate genes that, when upregulated, improve the growth

L358-361. "much higher" overclaims the impact of a single gene from a systems and control-theory perspective.

Response: The line has been edited.

[illegible] the optimal expression level (corresponding to maximum fitness) in the absence of overexpression costs is expected to be higher than the expression optimum in the presence of

L454-455. grammar error "is reflects"

Response: The line has been edited.

library of coding mutations with different expression strengths to elucidate the evolutionary constraints on coding and non-coding sequences. In these contexts, modifying expression levels reflects the potential effects of mutations on expression levels. For example, in a mutational

L459-464. Provide a brief physiological explanation for the contrasting fitness effect of dfrB1 mutations.

R: The section has been edited to add a physiological explanation.

[illegible] the other (Cisneros *et al*, 2023). At the physiological scale, DHFR function is coupled to thymidylate synthase (TYMS), whose activity must remain below that of DHFR to maintain folate balance (Schober *et al*, 2019). At low expression of *dfrB1*, TYMS activity may exceed DHFR's especially for loss of function variants, leading to the depletion of reduced folates. So mutations that increase the abundance of such variants are beneficial. At higher expression on the other hand, folate imbalance is likely alleviated. Therefore, further increases in protein abundance beyond this point are expected to be costly and hence the same coding mutations may now have different fitness effects.

L469-487. I was a bit lost when reading this paragraph. To avoid that, the author should make the point "fitness effects of coding mutations depend not only on their biochemical properties but also on the level of gene expression" clear at the beginning of this paragraph.

Also, the fact that highly expressed genes evolve more slowly likely attribute to both gene essentiality and avoiding translational/folding errors. Personally, I wouldn't use "challenge" here. I am also not sure if "highly expressed genes evolve more slowly" is a relevant example for the regulatory-coding epistasis, unlike the promoter-glutamate dehydrogenase dependence.

Response: The section has been edited. The reviewer is right in pointing out slow coding sequence evolution of highly expressed genes may be due to purifying selection or prevention of translational errors. However, regulatory-coding epistasis is a fitness-level phenomenon that results from several molecular causes, including the ones that drive the slow evolution of overexpressed genes.

Genomic analyses lend further support to the dependence of fitness effects of coding mutations on the expression levels. Evolutionary rate studies have shown that highly expressed genes tend to evolve more slowly than genes expressed at lower levels (Martincorena *et al*, 2012; Stefani & Dobson, 2003; Jayaraman *et al*, 2022; Bédard *et al*, 2022; Drummond *et al*, 2005). Initially, this was attributed to the essential nature of highly expressed genes and their centrality in protein-protein interaction networks, making mutations in them highly deleterious (Pál *et al*, 2001). However, subsequent work has proposed that high expression selects for coding sequences that are robust to translational errors, favoring amino acid choices that reduce misfolding risks. Accordingly, the relationship between expression and evolutionary rate reflects selection for protein stability and translational accuracy, rather than functional importance per se (Drummond *et al*, 2005). Experimental support for the misfolding avoidance hypothesis came from a study in yeast using GFP and URA3 as model proteins. This work showed that the fitness effects of coding mutations depend not only on their biochemical properties but also on the level of gene expression and the environmental context. Mutations that might be tolerated at low expression could become harmful when expression is high, particularly under stress or when misfolded proteins accumulate (Wu *et al*, 2022). Accordingly, overexpression may aggravate the deleterious effects of some coding variants. In this view, expression level modulates the deleteriousness of amino acid substitutions even over evolutionary timescales. However, it must be noted that the DMS study carried out on *dfrB1* in *E. coli* did not make the same observations (Cisneros *et al*, 2023), suggesting that these effects could depend on the properties of the proteins studied.

L553. "core" promoter region or promoter proximal element?

Response: The line has been edited.

Lupski, 2015). Recent estimates indicate that 90% of mutations in enhancers are neutral, and approximately 30% of mutations in core promoter regions are deleterious (Di *et al*, 2025).

L562-565. Briefly explain the basis (statistical/functional) of this study's interpretation.

Response: An explanation for this study has been added.

A genome-wide association study reported in 2004 was one of the first to study the combined effects of regulatory and coding variation in humans. Using the genotype and gene expression data obtained from about 200 individuals, the findings of this study showed that about 18% of the coding mutations exhibited phenotypes that were significantly dependent on the *cis*-regulatory variant (Dimas *et al*, 2008). Functionally, the findings of this study highlight that *cis*-regulatory variation can mask or amplify the phenotypic effects of protein coding variants.

L565-568. Similar to my question about "highly expressed genes evolve more slowly", is the observation here due to gene essentiality or regulatory-coding epistasis?

Response: The reviewer is correct in pointing out that the slow coding sequence evolution of highly expressed genes may be due to gene essentiality. However, in highly expressed genes, coding mutations have higher functional and energetic costs. As a result, the fitness effects of coding variants depend on the regulatory context, leading to stronger purifying selection against coding mutations specifically in highly expressed genes.

L622-624. The modes of action of these non-coding mutations are described in PMID: 22832162. Cite this reference for a mechanistic explanation.

Response: Thanks, this reference has been added.

mutation often led to the most deleterious combinatorial outcome, a pattern explained by the nonlinear mapping from expression to fitness (as illustrated in **Fig 2C**) (Chou *et al*, 2014; Chou & Marx, 2012).

L701-704. Cite two relevant DMS examples for expression-altering mutations doi:
10.1101/gr.260182.119
10.7554/eLife.64543

Response: Thanks, these references have been added.

While mutations affecting activity are well known to produce large fitness effects (Gerasimavicius *et al*, 2022), less is understood about the effect sizes of regulatory or expression-altering mutations (Kuo *et al*, 2020; Lagator *et al*, 2022). A recent work shows that only a small fractionof mutations, if any, in a yeast promoter provide adaptive benefits because of the non-linear relationship between fitness and expression level (Aubé *et al*, 2025). However, it is unknown how coding mutations may interact with such a regulatory landscape. Overall, regulatory-coding epistasis reshapes the adaptive landscape by modulating the distribution of fitness effects and altering environment-dependence of mutational effects, that complicates the prediction of evolutionary trajectories. However, most evidence comes from a few studies, with limited exploration of the full combinatorial space in experimentally tractable contexts. In the following perspective, we consider what these findings imply for how we interpret genetic variation, predict evolutionary outcomes, and design future studies to uncover general principles of genome evolution. evolution, affecting not just the outcomes of selection but the rules by which genetic variation is interpreted and acted upon.

Fig. 1A. The stacked histogram can be plotted in a more intuitive manner. Particularly, I found using diagonal -line boxes to indicate ranges perplexing. I had to read the legend twice to get the meaning of fn and fs.

Response: Figure 1 has been changed.

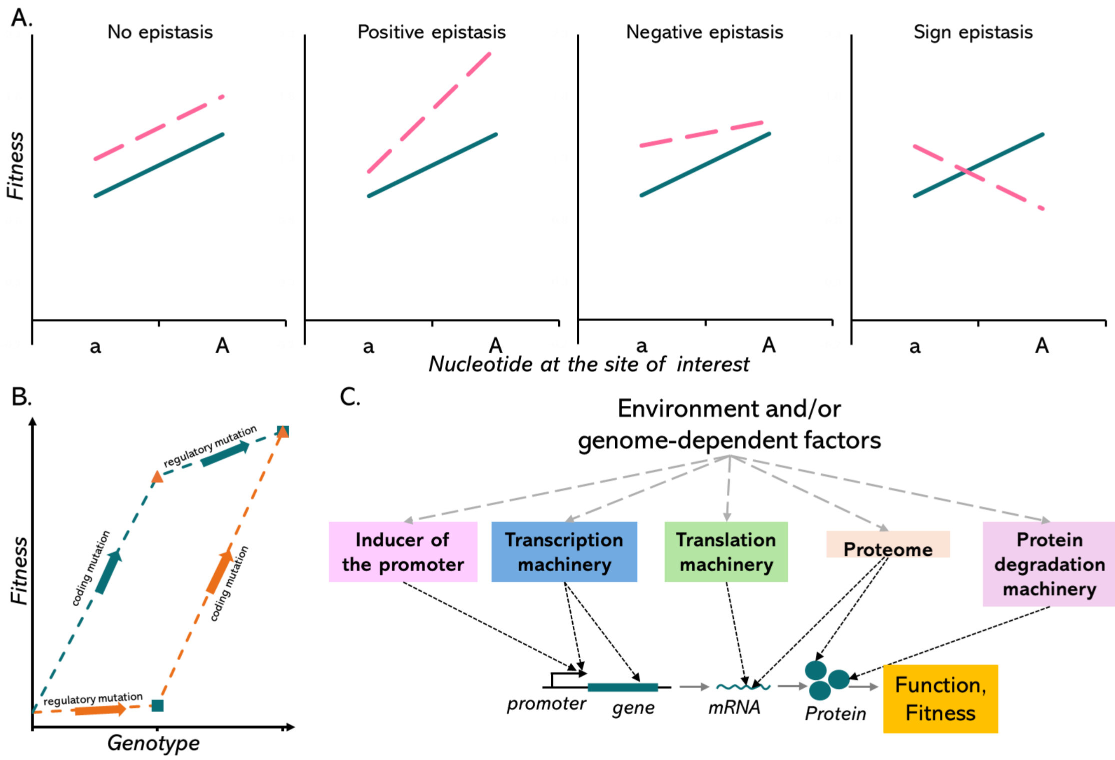

Fig 1C should include the post-transcriptional regulation step as it is particularly important for eukaryotic gene expression.

Response: Thanks for this comment. While at the molecular level, processes like transcription and translation are highly complex with several processes like post-transcriptional modifications, post-translational modifications, chromatin remodeling, etc., the purpose of this figure is to give a bird's eye view of the effects of changes in the genome and environment. We have edited the caption to specify that this figure is not all-encompassing.

Fig 1. The fitness effects of non-coding mutations depend on the genetic background and the environment. (A) This cartoon illustrates how the fitness effect of a mutation (a→A in a specific site) changes across two genetic backgrounds: *b* (solid green line) and *B* (dashed pink line). In the no epistasis case, the fitness effect of the mutation is identical in both backgrounds. In the positive epistasis case, the mutation's effect is larger in background *B* than in *b*. But when there is negative epistasis, the mutation still increases fitness, but its effect is smaller in *B* than in *b*. Sign epistasis represents an extreme scenario in which the background changes the direction of effect itself: the mutation is beneficial in background *b* but not in *B*. In general, sign epistasis produces a fitness effect whose magnitude is smaller than that observed in no epistasis condition. (B) Examples of epistasis between pairs of mutations are well documented. Here, we illustrate a case of epistasis between a regulatory and a coding mutation (inspired by Brown *et. al.*) (Brown *et al*, 2009). The wild-type genotype has the lowest fitness. A regulatory mutation (blue square) alone provides only a small fitness benefit, while a coding mutation (orange triangle) alone leads to a larger increase in fitness. However, when the regulatory mutation occurs on the background of the coding mutation, its fitness effect is much greater than when it occurs on the wild-type background. This suggests a form of positive epistasis, where the regulatory mutation is more beneficial in the presence of the coding change. In an adaptive context, this implies that the coding mutation is likely fixed first, followed by the regulatory mutation. (C) While the fitness effects of mutations depend on both the genetic background and the environment, the underlying causes often involve one or more molecular changes. Genomic and environmental alterations can affect the abundance and composition of RNA polymerases, ribosomes, amino acids, and the proteome, including post-transcriptional and post-translational regulators depending on the organism's complexity, protein degradation machinery, and other interacting proteins, as well as how the cell imports chemicals from its surroundings. Together, these changes influence promoter activity, transcription, translation, protein abundance, protein–protein interactions, and ultimately, cellular function and fitness.

Fig. 2C. The magnitude of the fitness burden is too large.

R: Thanks. Figure 2C has been edited.

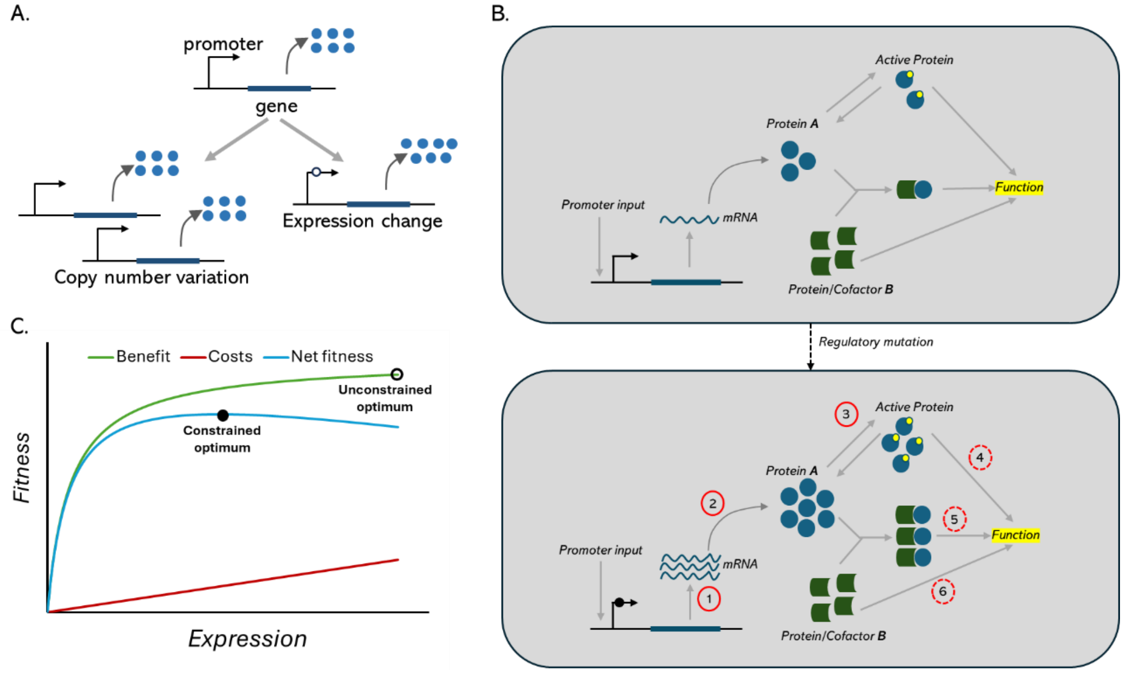

Dear Dr. Venkataraman,

Thank you for the submission of your revised review article to EMBO Reports. I sincerely apologize for the delay in handling it, but we have received the two enclosed reports on it and as you will see, both referees support publication after some minor revisions.

From the editorial side, there are a few points that we kindly ask you to address:

- Please add a 'Disclosure and competing interests statement'.

- Information on funding needs to be part of the Acknowledgments section. The 'Funding' section heading is not necessary.

- Please provide a short paragraph called "Box 1: In need of answers", that allows you to describe open questions in the field in the form of a bullet points list.

Once the final version of your Review article is in, I will forward your figures to the team of graphics designers that we work with. As soon as the figures have been approved, we can proceed with publication.

Kind regards,

Martina

Martina Rembold, PhD
Senior Editor
EMBO reports

==================

Referee #1:

The manuscript has been improved.

Referee #2:

The content, particularly graphic presentation in the updated Figure 1, has improved significantly, which addresses my main concern raised previously. Here, I only have minor comments, as well as suggestions that aim to improve sentence structure and clarity. I encourage the authors to polish and tighten sentences through the manuscript.

> Minor comments:
L67-69: If empirical data of every mutational effect are gathered across multiple environments, then what is left is just quantitative interpretation rather than prediction.

> Suggestion for sentence clarity:
L50-52: Therefore, their effects are difficult to generalize, raising the key question: how do regulatory mutation-induced changes in protein abundance translate into fitness changes?

L58-61: Simplify these sentences, which the main idea is "Mutational effects are context-dependent."

L96: Rephrase, "not all proteins with destabilizing mutations increase disease risks."

L109-110: This sentence is really confusing, with lots of "and." What are the two levels here?

L110-113: Simplify. How do phenomena mentioned here relate to the two levels described just before?

L114-116: Understanding protein evolution therefore requires an integrated approach accounting for regulatory architecture governing gene expression, the structural and functional effects of coding mutations, and the genomic and environmental context shaping their interplay.

L120-121: delete "adaptive" and "by constraining or facilitating adaptation."

Figure legends: All of them are quite long.

Point-by-point responses to reviewer comments:

We are grateful for the feedback and suggestions received from the reviewers, and have incorporated the necessary changes as detailed below.

Referee #1:

The manuscript has been improved.

Referee #2:

The content, particularly the graphic presentation in the updated Figure 1, has improved significantly, which addresses my main concern raised previously. Here, I only have minor comments, as well as suggestions that aim to improve sentence structure and clarity. I encourage the authors to polish and tighten sentences through the manuscript.

> Minor comments:

L67-69: If empirical data of every mutational effect are gathered across multiple environments, then what is left is just quantitative interpretation rather than prediction.

Response: We have altered the sentence to clarify this point.

> mutations often unpredictable from the effect of single ones (Sandhu *et al*, 2025). Therefore, in the absence of a mechanistic understanding of how epistasis operates at the cellular level, fully describing evolution requires that the fitness effect of every mutation in every possible genotype is measured in as many environments as possible. However, as the genome length increases, the number of possible mutations increases, and the number of possible genotypic combinations grows exponentially, far exceeding what can be systematically studied in the laboratory or even simulated computationally.

> Suggestion for sentence clarity:

L50-52: Therefore, their effects are difficult to generalize, raising the key question: how do regulatory mutation-induced changes in protein abundance translate into fitness changes?

Response: We have altered this sentence according to the suggestion.

> 2022; Wittkopp *et al*, 2008; McManus *et al*, 2010), and in some cases in multicellular organisms, cell-dependent (Scacheri & Scacheri, 2015). Therefore, their effects are difficult to generalize, raising the key question: how do regulatory mutation-induced changes in protein abundance translate into fitness changes? It is well established that insufficient and excessive protein levels can be detrimental (Bolognesi & Lehner, 2018). However, we currently lack a quantitative understanding of the fitness effects and evolutionary contingencies associated with under- or overexpression, as the underlying causes are poorly characterized at a general level across genes and conditions (Moriya, 2015).

L58-61: Simplify these sentences, which the main idea is "Mutational effects are context-dependent."

Response: We have altered the sentence to simplify it.

Understanding evolutionary changes is also complex due to the fact that the fitness effects of a mutation depend on the broader genetic and environmental context in which they arise. Epistasis, or the interaction between genetic loci, is pervasive (Bateson & Mendel, 1909). Epistasis can occur within a single coding region (Starr & Thornton, 2016), between distinct coding loci (Johnson *et al*, 2023), within non-coding regions (Kuo *et al*, 2020; Ang *et al*, 2023; Bernet & Elena, 2015), between non-coding regions (McQueen *et al*, 2025), or across regulatory and coding elements (Lagator *et al*, 2017). Any of these forms of epistasis alters mutational effects in non-additive ways (**Fig 1A**), making the fitness effects of combinations of

L96: Rephrase, "not all proteins with destabilizing mutations increase disease risks."

Response: We have altered the sentence according to the suggestion.

However, stability, or such a protein property, alone cannot fully explain evolutionary outcomes. For example, a protein-destabilizing mutation does not always lead to disease. This is partly because mutations that affect a protein's structure or function arise in different

L109-110: This sentence is really confusing, with lots of "and." What are the two levels here?

Response: We have altered the sentence to clarify the idea.

Epistatic relationships are additionally complicated by genotype-by-environment (GxE) interactions, which modulate the fitness outcomes of mutations, largely based on the need of the protein they affect (Johannsen, 1911). The environment can act at two levels to modify the fitness of genotypes (Aubé & Landry, 2024). It can alter the mapping between activity, abundance and function, or, function and fitness. Generally, changing environments could alter regulation dynamics (Penner-Goeke & Binder, 2024) and hence protein abundance (Munro *et al*, 2024), metabolic state of the cell (Mallard *et al*, 2018) and through that, modify the fitness-optimal protein abundance and activity (Dekel & Alon, 2005). Understanding protein evolution therefore requires an integrated approach accounting for regulatory architecture governing gene expression, the structural and functional effects of coding mutations, and the genomic and environmental context shaping their interplay (**Fig 1C**).

In this review, we examine how variation in protein abundance, together with changes in structure and activity, shapes the evolutionary dynamics of proteins. In this Darwinian context,

L110-113: Simplify. How do phenomena mentioned here relate to the two levels described just before?

Response: We have altered the sentence to a simpler one.

Epistatic relationships are additionally complicated by genotype-by-environment (GxE) interactions, which modulate the fitness outcomes of mutations, largely based on the need of the protein they affect (Johannsen, 1911). The environment can act at two levels to modify the fitness of genotypes (Aubé & Landry, 2024). It can alter the mapping between activity, abundance and function, or, function and fitness. Generally, changing environments could alter regulation dynamics (Penner-Goeke & Binder, 2024) and hence protein abundance (Munro *et al*, 2024), metabolic state of the cell (Mallard *et al*, 2018) and through that, modify the fitness-optimal protein abundance and activity (Dekel & Alon, 2005). Understanding protein evolution therefore requires an integrated approach accounting for regulatory architecture governing gene expression, the structural and functional effects of coding mutations, and the genomic and environmental context shaping their interplay (**Fig 1C**).

In this review, we examine how variation in protein abundance, together with changes in structure and activity, shapes the evolutionary dynamics of proteins. In this Darwinian context,

L114-116: Understanding protein evolution therefore requires an integrated approach accounting for regulatory architecture governing gene expression, the structural and functional effects of coding mutations, and the genomic and environmental context shaping their interplay.

Response: We have altered the sentence according to the suggestion.

Epistatic relationships are additionally complicated by genotype-by-environment (GxE) interactions, which modulate the fitness outcomes of mutations, largely based on the need of the protein they affect (Johannsen, 1911). The environment can act at two levels to modify the fitness of genotypes (Aubé & Landry, 2024). It can alter the mapping between activity, abundance and function, or, function and fitness. Generally, changing environments could alter regulation dynamics (Penner-Goeke & Binder, 2024) and hence protein abundance (Munro *et al*, 2024), metabolic state of the cell (Mallard *et al*, 2018) and through that, modify the fitness-optimal protein abundance and activity (Dekel & Alon, 2005). Understanding protein evolution therefore requires an integrated approach accounting for regulatory architecture governing gene expression, the structural and functional effects of coding mutations, and the genomic and environmental context shaping their interplay (**Fig 1C**).

In this review, we examine how variation in protein abundance, together with changes in structure and activity, shapes the evolutionary dynamics of proteins. In this Darwinian context,

L120-121: delete "adaptive" and "by constraining or facilitating adaptation."

Response: The edits have been made.

Epistatic relationships are additionally complicated by genotype-by-environment (GxE) interactions, which modulate the fitness outcomes of mutations, largely based on the need of the protein they affect (Johannsen, 1911). The environment can act at two levels to modify the fitness of genotypes (Aubé & Landry, 2024). It can alter the mapping between activity, abundance and function, or, function and fitness. Generally, changing environments could alter regulation dynamics (Penner-Goeke & Binder, 2024) and hence protein abundance (Munro *et al*, 2024), metabolic state of the cell (Mallard *et al*, 2018) and through that, modify the fitness-optimal protein abundance and activity (Dekel & Alon, 2005). Understanding protein evolution therefore requires an integrated approach accounting for regulatory architecture governing gene expression, the structural and functional effects of coding mutations, and the genomic and environmental context shaping their interplay (**Fig 1C**).

In this review, we examine how variation in protein abundance, together with changes in structure and activity, shapes the evolutionary dynamics of proteins. In this Darwinian context,

Figure legends: All of them are quite long.

Response: We have shortened three legends as much as possible while not diminishing their importance.

Legends

Fig 1. The fitness effects of non-coding mutations depend on the genetic background and the environment. (A) This cartoon illustrates how the fitness effect of a mutation (a→A in a specific site) changes across two genetic backgrounds: *b* (solid green line) and *B* (dashed pink line). With no epistasis, the mutation has the same effect in both backgrounds. In positive epistasis, the effect is larger in B than in b; in negative epistasis, it remains beneficial but smaller in B. Sign epistasis represents an extreme case where the mutation is beneficial in b but not in B. (B) Example of epistasis between regulatory and coding mutations (inspired by *Brown et al., 2009 (Brown et al. 2009))*. The regulatory mutation alone provides a small benefit, while the coding mutation gives a larger benefit. However, the regulatory mutation becomes much more beneficial when combined with the coding mutation, illustrating positive epistasis and suggesting sequential fixation of coding followed by regulatory changes. (C) While the fitness effects of mutations depend on both the genetic background and the environment, the underlying causes often involve one or more molecular changes. Genomic and environmental alterations can affect the abundance and composition of RNA polymerases, ribosomes, amino acids, and the proteome, including post-transcriptional and post-translational regulators depending on the organism's complexity, protein degradation machinery, and other interacting proteins, as well as how the cell imports chemicals from its surroundings. Together, these changes influence promoter activity, transcription, translation, protein abundance, protein–protein interactions, and ultimately, cellular function and fitness.

Fig 2. Protein abundance, costs, and evolutionary constraints on expression. (A) A promoter–gene pair producing 6 proteins yields 12 upon copy number doubling. In contrast, promoter mutations alter expression independently of copy number, enabling finer, non-integral control of protein abundance. (B) The top panel illustrates the production of a protein and the downstream processes it is involved in, in a cell. A promoter mutation (denoted by a black circle) that increases expression can impose significant costs on the cell. It leads to increased transcription ①, which consumes energy and RNA nucleotides and polymerases, and increased translation ②, which demands additional energy, ribosomes, and amino acids. Once translated, the protein exists in both active and inactive forms, and the transition between these states is energetically demanding ③. These are the direct costs of overexpression (indicated by numbers in continuous red circles). The active form performs its primary cellular function, while the inactive form interacts with another protein to carry out a secondary role ④. Since protein A is overproduced, it could sequester the cofactors that it binds with to perform cellular function ⑤. This sequestration of the cofactor could result in a decrease in the function that the cofactor individually performs ⑥. The excess accumulation of the different forms of the protein and their associated complexes can overwhelm the cell's protein quality control systems, disrupting protein homeostasis. Additionally, the downstream stoichiometric changes may have fitness costs. These types of non-energetic, indirect costs are indicated in discontinuous circles in the figure. (C) The net fitness resulting from overexpression reflects the balance between its benefits and costs. When fitness effects saturate with expression, as shown by the green curve, and costs function is convex, as shown by the red line, the resulting net fitness follows the blue curve. Without any cost of overexpression, the optimal expression level would be higher than when such costs are present.

Fig 3. Regulatory-coding epistasis and its effect on disease risk. (A) The fitness effects of a library of single amino acid mutations in yeast Hsp90 were measured across different expression levels. The figure (based on the average fitness effect data from Jiang *et. al. (Jiang et al. 2013)* shows the distribution of fitness effects (DFE), which describes the range and likelihood of fitness consequences of new mutations (*s*). The DFE clearly shifts with expression: higher expression levels, by increasing transcription, buffer the deleterious effects of some mutations but also reduce the benefits of others, resulting in a narrower distribution. (B) A similar, more comprehensive deep mutational scanning study of the *dfrB1* gene in *E. coli* further highlights the influence of expression on mutational effects. The figure (based on the average fitness effect data from Cisneros *et. al. (Cisneros et al. 2023)* compares fitness effects at high or intermediate expression levels (promoter activity) with fitness at low expression. Increased expression buffers mildly deleterious mutations but also reveals the costs of overexpression, as beneficial mutations show reduced fitness gains at higher expression levels. (C) This example (inspired by Castel *et al., 2018 (Castel et al. 2018)*) illustrates how regulatory and coding mutations interact to produce disease in a heterogeneous population. In the wild type, the gene is not transcribed, resulting in no protein and low disease penetrance. A homozygous regulatory mutation activates transcription, producing protein and increasing penetrance, similar to a homozygous deleterious coding mutation that is not expressed. However, when both mutations are homozygous, expression of the mutant protein causes disease in most individuals. Thus, regulatory mutations can non-additively modulate the penetrance of disease-causing coding variants.

2nd Revision - Editorial Decision **29th Apr 2026**

Dr. Pavithra Venkataraman
Université Laval
Biochemistry, Microbiology and Bioinformatics
Canada

Dear Dr. Venkataraman,

I am pleased to inform you that your review article has been accepted for publication in EMBO reports.
Your figures are being redrawn by a team of graphics designers we work with. Within approx. 10 working days you will be contacted with a draft of the figures for your approval.

Your manuscript will then be processed for publication by EMBO Press. It will be copy edited and you will receive page proofs prior to publication. Please note that you will be contacted by Springer Nature Author Services to complete licensing information.

Should you be planning a Press Release on your article, please get in contact with embo_production@springernature.com as early as possible in order to coordinate publication and release dates.

If you have any questions, please do not hesitate to contact the Editorial Office. Thank you for your contribution to EMBO Reports.

Kind regards,

Martina

Martina Rembold, PhD
Senior Editor
EMBO reports

>>> Please note that it is EMBO Reports policy for the transcript of the editorial process (containing referee reports and your response letter) to be published as an online supplement to each paper. If you do NOT want this, you will need to inform the Editorial Office via email immediately. More information is available here: https://link.springer.com/partners/embo-press/editorial-policies#Peer%20review
